# Changes of drug pharmacokinetics mediated by downregulation of kidney organic cation transporters Mate1 and Oct2 in a rat model of hyperuricemia

**DOI:** 10.1371/journal.pone.0214862

**Published:** 2019-04-05

**Authors:** Kei Nishizawa, Noriaki Yoda, Fumi Morokado, Hisakazu Komori, Takeo Nakanishi, Ikumi Tamai

**Affiliations:** 1 Faculty of Pharmaceutical Sciences, Institute of Medical, Pharmaceutical and Health Sciences, Kanazawa University, Kanazawa, Japan; 2 Department of Drug Metabolism and Pharmacokinetics, Tokushima Research Institute, Otsuka Pharmaceutical Co., Ltd., Tokushima, Japan; University Medical Center Utrecht, NETHERLANDS

## Abstract

The effects of hyperuricemia on the expression of kidney drug transporters and on the pharmacokinetics of several substrate drugs were examined. We first established a rat model of hyperuricemia without marked symptoms of chronic kidney failure by 10-day co-administration of oxonic acid (uricase inhibitor) and adenine (biosynthetic precursor of uric acid). These hyperuricemic rats showed plasma uric acid concentrations of up to 6 mg/dL, which is similar to the serum uric acid level in hyperuricemic humans, with little change of inulin clearance. The mRNA levels of multidrug and toxin extrusion 1 (Mate1, Slc47a1), organic anion transporter 1 (Oat1, Slc22a6), organic cation transporter 2 (Oct2, Slc22a2), urate transporter 1 (Urat1, Slc22a12) and peptide transporter 1 (Pept1, Slc15a1) were significantly decreased in kidney of hyperuricemic rats. Since Oct2, Mate1 and Oat1 are important for renal drug elimination, we next investigated whether the pharmacokinetics of their substrates, metformin, cephalexin and creatinine, were altered. The plasma concentration of metformin was not affected, while its kidney tissue accumulation was significantly increased. The plasma concentration and kidney tissue accumulation of cephalexin and the plasma concentration of creatinine were also increased. Furthermore, the protein expression of kidney Mate1 was decreased in hyperuricemic rats. Accordingly, although multiple factors may influence renal handling of these drugs, these observations can be accounted for, at least in part, by downregulation of Mate1-mediated apical efflux from tubular cells and Oct2-mediated basolateral uptake. Our results suggest that hyperuricemia could alter the disposition of drugs that are substrates of Mate1 and/or Oct2.

## Introduction

Reduced liver or renal function due to chronic disease can result in altered expression of drug-metabolizing enzymes and transporters [1], and dose adjustment of some drugs is recommended in patients who have reduced liver and/or kidney functions to minimize the risk of adverse events [2]. For example, metformin is used to treat type II diabetes, but it can cause lactic acidosis [3]; therefore, because metformin is exclusively eliminated in urine [4], patients’ renal function should be carefully monitored.

Hyperuricemia, defined as a serum uric acid level higher than 7 mg/dL [5], is associated with several common diseases, including cardiovascular disease, chronic kidney disease, hypertension, and gout [6–8], which often require long-term drug treatment. Uric acid levels in the body are regulated by various transporters in kidney and intestine, as well as by the biosynthetic enzyme xanthine oxidase [9, 10], so drugs that interact with these transporters and enzymes may disturb uric acid homeostasis. For example, we have reported that several clinically used drugs, including angiotensin II receptor blockers, salicylic acid, and glucose-lowering sodium glucose cotransporter 2 (SGLT2, SLC5A2) inhibitors, affect uric acid disposition through interactions at transporters in the kidney and intestine [11–14]. We also reported that hyperuricemia causes decreased expression of breast cancer resistance protein (BCRP, ABCG2) transporter in the plasma membrane of vascular endothelial cells, resulting in a nonlinear increase of cellular uric acid accumulation [15]. Changes in transporter expression can also alter the pharmacokinetics of substrate drugs. For example, in hyperuricemic rats, expression of renal organic ion transporters organic anion transporter (Oat) 1 (Slc22a6), Oat3 (Slc22a8) and organic cation transporter (Oct) 2 (Slc22a2) is decreased, resulting in reduced clearance of methotrexate and cimetidine [16]. Therefore, it is important to establish in detail the effects of hyperuricemia on transporter expression and drug pharmacokinetics.

So far, animal models of hyperuricemia have generally been prepared by administration of oxonic acid, a uricase inhibitor, to block metabolism of uric acid to allantoin [17]. Administration of adenine, a biosynthetic precursor of uric acid, is also effective to increase the plasma concentration of uric acid, but prolonged administration of adenine causes various symptoms of chronic kidney failure [18]. Accordingly, it is not easy to prepare hyperuricemic model animals without impairment of kidney functions.

Therefore, in the present study, we first established a rat model of hyperuricemia with minimal changes of kidney functions by means of combined short-term administration of oxonic acid and adenine, in order to examine the specific effects of hyperuricemia on the expression of drug transporters in the kidney and on the disposition of substrate drugs of the affected transporters. Among transporters whose expression was reduced, we focused on multidrug and toxin extrusion 1 (Mate1, Slc47a1), Oct2 and Oat1, since they are important for drug elimination in the kidney, and investigated whether their downregulation alters the pharmacokinetics of their substrates metformin, cephalexin and creatinine, which are exclusively excreted intact into urine.

## Materials and methods

### Materials

Uric acid, creatinine and creatinine-d_3_ were purchased from Wako Pure Chemical Industries (Osaka, Japan). Oxonic acid potassium salt, metformin and cephalexin were purchased from Sigma Aldrich (St. Louis, MO). Other reagents and solvents were of analytical grade.

### Animals

Seven-week-old male Wistar rats were purchased from Sankyo Labo Service (Tokyo, Japan). All animal studies were approved by the Committee of Kanazawa University for the Care and Use of Laboratory Animals and were performed in accordance with its guidelines (AP-143148). Rats were housed two to four per cage with free access to water and a commercial animal chow throughout the acclimatization and experimental periods; they were maintained on a 12-hour dark/light cycle in an air-controlled room (temperature, 24.0 ± 10°C; humidity, 55 ± 5%).

Hyperuricemic rats were prepared by oral administration of adenine (0.1 g/kg) and oxonic acid potassium salt (1.5 g/kg) suspended in 0.5% (w/v) methylcellulose solution daily for 10 days. Vehicle control rats received an equal volume per body weight of 0.5% (w/v) methylcellulose solution.

### In vivo animal study

Pharmacokinetic studies were conducted 10 days after starting administration of oxonic acid and adenine (Day 10). Rats were anesthetized with pentobarbital, and the bladder was cannulated with polyethylene tubing (inside diameter 0.5 mm, outside diameter 0.8 mm). The rats were given 30 mg/kg metformin, 1 or 10 mg/kg cephalexin or 100 mg/kg inulin intravenously via the femoral vein. Blood was drawn through the jugular vein, and plasma was obtained by centrifugation at 3,000 rpm for 10 min at 4°C. Urine was collected at the designated times. Rats were sacrificed 4 h after administration of each compound by cutting the inferior vena cava under deep anesthesia, and the kidneys were isolated. All samples were stored at -80°C until measurement.

### Quantification of mRNA expression

Total RNA was isolated from the kidneys collected on Day 10 using RNAiso Plus (TaKaRa Bio, Shiga, Japan) and reverse-transcribed into cDNA using M-MLV Reverse Transcriptase (Promega, Madison, WI). Quantitative RT-PCR was performed using FastStart Universal SYBR Green Master (Roche Applied Science, Indianapolis, IN) and Mx3000P QPCR system (Agilent Technologies, Santa Clara, CA). Primers are shown in S1 Table. Expression was estimated by the -ΔΔCt method and normalized to that of glyceraldehyde-3-phosphate dehydrogenase (Gapdh) [19].

### Analytical methods

If necessary, plasma and urine were diluted 10- to 100-fold and 10- to 10000-fold with saline, respectively, for high-performance liquid chromatography (HPLC) or liquid chromatography/tandem mass spectrometry (LC-MS/MS) measurement. For quantification of tissue concentrations, the tissues were homogenized in 4 times the tissue weight of saline. To denature proteins, all biological samples were mixed with 9 volumes of acetonitrile containing internal standard (for metformin: 2 μM rosuvastatin, for cephalexin: 10 μM amoxicillin, for creatinine: 100 ng/mL creatinine-d_3_) for LC-MS/MS assay. Prepared samples were mixed for 1 min and centrifuged at 15,000 rpm for 5 min at 4°C. The resultant supernatant was used for LC-MS/MS analysis. The LC-MS/MS system consisted of a triple-quadrupole mass spectrometer (API 3200^TM^, SCIEX, Foster City, CA) coupled with an ultrafast liquid chromatography system (LC-20AD, Shimadzu, Kyoto, Japan). Chromatography was performed using a CAPCELL PAK C18 HPLC packed column (4.6 × 100 mm, particle size 5 μm, Shiseido, Tokyo, Japan) prewarmed to 40°C for quantification of metformin and cephalexin, or an Atlantis HILIC Silica (2.1 × 150 mm, particle size 5 μm, Waters, Milford, MA) prewarmed to 40°C for creatinine. For metformin, the mobile phase consisted of 0.1% formic acid and acetonitrile (30 : 70), and the flow rate was 0.4 mL/min. For cephalexin, the mobile phase consisted of 0.1% formic acid and acetonitrile (77 : 23) containing 0.1% formic acid, and the flow rate was 0.7 mL/min. For creatinine, the mobile phase consisted of 0.1% formic acid and acetonitrile (13 : 87) containing 0.1% formic acid, and the flow rate was 0.5 mL/min. The following multiple reaction monitoring transitions ([M+H]^+^, *m/z*, Q1 → Q3) were selected: metformin (*m/z*, 130.1 → 60), rosuvastatin (*m/z*, 482.1 → 258), cephalexin (*m/z*, 348.157 → 158), amoxicillin (*m/z*, 367.09 → 114), creatinine (*m/z*, 113.985 → 86.1) and creatinine-d_3_ (*m/z*, 117.1 → 89.2).

Extraction of uric acid from plasma was performed by adding 9 volumes of 10% perchlorate, stirring for 1 min, and centrifuging at 15,000 rpm for 5 min at 4°C. To the resultant supernatant was added an equal volume of 0.5% acetic acid for HPLC analysis. The HPLC system (Waters) was equipped with a solvent delivery system (Waters 2695) and a UV absorbance detector (Waters 2487/2690). Chromatography was performed using a Mightysil RP-18GP Aqua analytical column (4.6 mm × 250 mm, particle size 5 μm, Kanto Chemical, Tokyo, Japan) and the mobile phase was 0.5% acetic acid (isocratic, 1.0 mL/min). Uric acid was detected at 274 nm.

Inulin was measured by the anthrone method [20]. After adding 500 mL concentrated sulfuric acid to 200 mL distilled water and cooling the solution to room temperature, 1.4 g anthrone (Sigma Aldrich) was added to it at 56°C to prepare the anthrone reagent. An aliquot of the reagent (400 μL) was added to 10 μL of plasma or urine suitably diluted with saline. The tube containing the sample was immersed in ice-cold water, shaken well and then allowed to react for 10 min at 56°C. The vial was immersed in ice-cold water to stop the reaction. The absorbance at 636 nm was measured with a spectrophotometer (UVmini1240, Shimadzu).

### Pharmacokinetic analysis

The plasma concentration-time data were analyzed by non-compartmental analysis. The area under the plasma concentration-time curve (AUC_0-4_) was obtained by application of the trapezoidal rule from time 0 to 4 h. AUC from 0 to infinity (AUC_inf_) was estimated by extrapolation to infinity. Total clearance (CL_tot_) was estimated as dose (D) over AUC_inf_ (D/AUC_inf_), and renal clearance (CL_R_) was estimated as X_urine,0-4_/AUC_0-4_, where X_urine,0–4_ represents the cumulative amount of drug recovered in the urine from 0 to 4 h. The apparent tissue-to-plasma concentration ratio (K_p, kidney_) was obtained as the ratio of kidney concentration divided by plasma concentration at 4 h after administration.

### Western blot analysis

Kidney tissue was homogenized in RIPA buffer (50 mM Tris-HCl, pH 8.0, 150 mM NaCl, 1.0% (v/v) nonidet P-40, 0.1% sodium dodecyl sulfate (SDS) and 0.5% (w/v) sodium deoxycholate) containing 1% protease inhibitor cocktail (Nacalai Tesque, Kyoto, Japan) using Ultra-Turrax homogenizer (model: T-25, IKA, Staufen, Germany). The homogenates were centrifuged at 15,000 rpm for 15 min at 4°C, and the supernatants were collected. After the determination of protein concentrations using Bio-Rad protein assay dye reagent (Bio-Rad Laboratories, Hercules, CA), the samples were denatured at 95°C for 5 min. The samples were subjected to SDS-PAGE on 10% polyacrylamide gel. Proteins were electroblotted onto a PVDF membrane (Millipore, Bedford, MA). After blocking in 2% skim milk, the membrane was washed three times and treated with anti-MATE1 antibody (1:500, GeneTex, Irvine, CA) at 4°C overnight. The membrane was further washed three times and incubated with horseradish peroxidase-conjugated anti-rabbit IgG (1:3,000, Invitrogen, Carlsbad, CA) for 2 h at room temperature. After the detection of Mate1, the membrane was washed and treated with anti-GAPDH antibody (1:5,000, Proteintech, Chicago, IL) for 2 h at room temperature. The membrane was further washed three times and incubated with horseradish peroxidase-conjugated anti-mouse IgG (1:5,000, Invitrogen) for 2 h at room temperature. Bands were detected using Immunostar Zeta (Wako Pure Chemical Industries, Osaka, Japan), according to the manufacturer’s instructions. The density of bands was determined using NIH Image 1.52 (National Institutes of Health).

### Cell culture

HEK293 cells transfected with human MATE1 (HEK293/hMATE1) and vector alone (mock) were gifts from Dr. Inoue (Tokyo University of Pharmacy and Life Science). Cells were cultured in Dulbecco’s modified Eagle’s medium supplemented with 10% fetal bovine serum, 100 units/ml penicillin, 100 μg/ml streptomycin, 1 mM pyruvic acid and 400 μg/mL G418 at 37°C in a humidified atmosphere of 5% CO_2_ in air.

### Uptake of [^3^H]MPP^+^ by MATE1-expressing cells

The uptake experiment was conducted as described previously [21]. HEK293/mock and HEK293/hMATE1 cells were plated at a density of 3.0 × 10^5^ cells/well on 24-well plates and cultured for two days before an uptake assay. The cells were pre-incubated with 0.5 mL of transport medium (TM; 125 mM NaCl, 4.8 mM KCl, 1.2 mM KH_2_PO_4_, 1.2 mM CaCl_2_, 1.2 mM MgSO_4_, 5.6 mM D-glucose, and 25 mM HEPES, pH 7.4) containing 30 mM NH_4_Cl for 20 min at 37°C to establish an outwardly directed H^+^ gradient. Uptake was initiated by replacing 0.25 mL of TM containing 0.25 nM [^3^H]N-methyl-4-phenylpyridinium acetate (MPP^+^, 80 Ci/mmol, American Radiolabeled Chemicals, Inc, St. Louis, MO) in the absence or the presence of oxonic acid, adenine or uric acid. After 0.5 min incubation at 37°C, uptake was terminated by washing the cells three times with 0.5 mL of ice-cold TM, and the cells were solubilized in 0.25 mL of 0.01% (v/v) Triton-X100. Radioactivity was measured with a liquid scintillation counter (Hitachi Aloka Medical, Tokyo, Japan). Part of the lysate was used for the determination of total protein amount with Bio-Rad protein assay dye reagent. Uptake of MPP^+^ was expressed as a cell-to-medium ratio (μL/mg protein) obtained by dividing the uptake amount by the concentration of substrate in the TM. Percentages of control were calculated as the ratio of MATE1-mediated uptake of MPP^+^ that obtained after subtraction of the uptake of MPP^+^ by mock cells in compound treated groups to that in control group.

### Statistical analysis

Unpaired Student’s t-test was used to analyze differences between groups except for uptake study, and Dunnett's test was used for uptake study to compare the control group to groups treated with drugs. *p < 0.05 was considered statistically significant.

## Results

### Preparation of hyperuricemic rats

**Fig 1:**
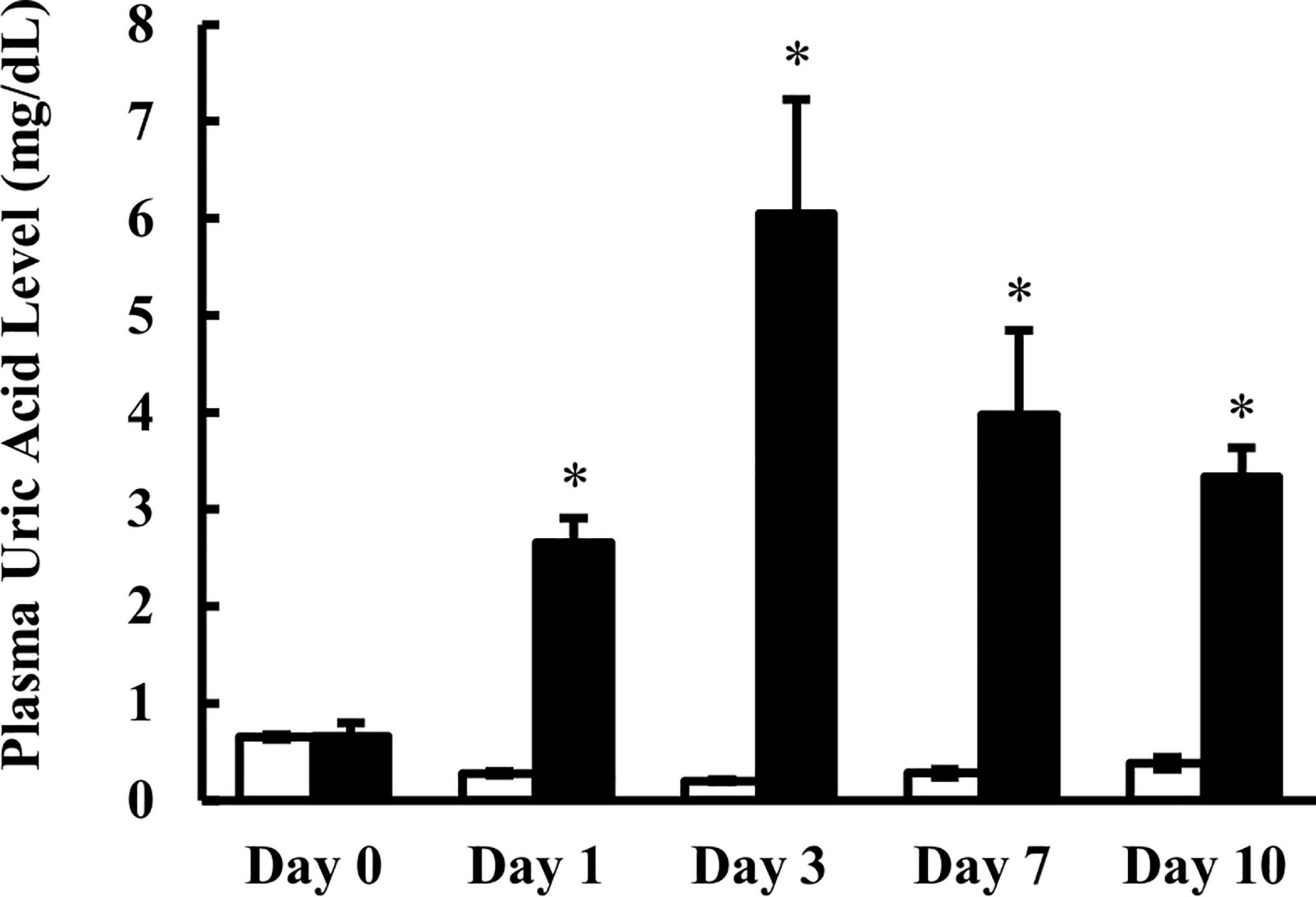
Plasma concentration of uric acid in control rats and oxonic acid plus adenine-treated rats during the 10-day administration period. Plasma was collected 3 h after oral administration of oxonic acid and adenine at doses of 1.5 g/kg and 0.1 g/kg, respectively. Open and closed columns represent control and oxonic acid and adenine-treated rats, respectively. Control rats were given vehicle alone (0.5% (w/v) methylcellulose solution). Data are presented as mean ± SEM (n = 3–7). *p < 0.05 vs. control rats.

Hyperuricemic model rats were prepared as described in Materials and Methods, which were aimed to establish a chronic model. As shown in Fig 1, plasma uric acid levels were increased from the day after the first oxonic acid/adenine administration, and reached a maximum of 6.05 mg/dL, which is close to the serum uric acid levels of hyperuricemic humans, on Day 3. During the 10-day administration period, hyperuricemic model rats showed plasma uric acid levels 8.7 to 30.1 times higher than those of control rats. To investigate the effect of the increased uric acid levels on kidney functions, markers were evaluated on Day 10. Plasma blood urea nitrogen (BUN) and creatinine levels were increased in hyperuricemic rats compared to normal rats, while inulin clearance was not significantly altered (Table 1).

**Table 1:** Plasma concentrations of BUN and creatinine and inulin clearance in control and hyperuricemic rats.

	Control rats			Hyperuricemic rats		
BUN (mg/dL)	15.4	±	1.2	34.2	±	1.5*
Plasma Creatinine (μM)	12.8	±	0.1	33.6	±	3.9*
Inulin Clearance (mL/min/kg)	6.72	±	0.46	5.36	±	0.77

Data are presented as mean ± SEM (n = 3–7).

*p < 0.05 vs. control rats.

### Changes in mRNA expression of kidney transporters

**Fig 2:**
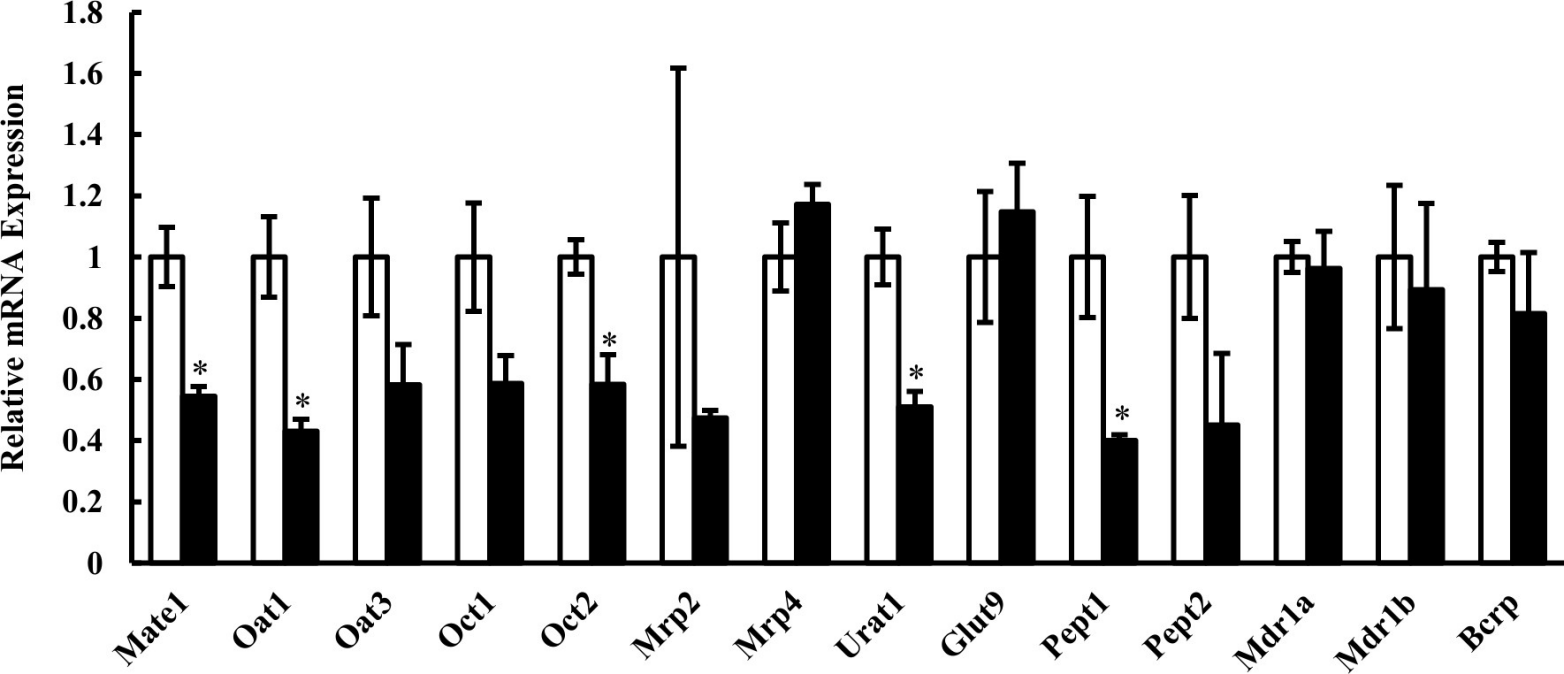
Relative mRNA expression levels of transporters in kidney of control and hyperuricemic rats. Relative mRNA levels normalized to Gapdh mRNA in hyperuricemic rats (closed columns) are shown as the ratio to those in control rats (open columns). Data are presented as mean ± SEM (n = 3–7). *p < 0.05 vs. control rats.

Next, the effects of hyperuricemia on the mRNA levels of kidney transporters on Day 10 were investigated. Among the 14 transporters examined, mRNA levels of Mate1, Oat1, Oct2, urate transporter 1 (Urat1, Slc22a12) and peptide transporter (Pept) 1 (Slc15a1) were significantly lower in hyperuricemic rats than in control rats (Fig 2). Since Mate1, Oct2 and Oat1 play central roles in drug elimination from the kidney, we examined whether the expression changes of these transporters altered the pharmacokinetics of their substrates.

### Pharmacokinetic changes in metformin, cephalexin and creatinine

**Fig 3:**
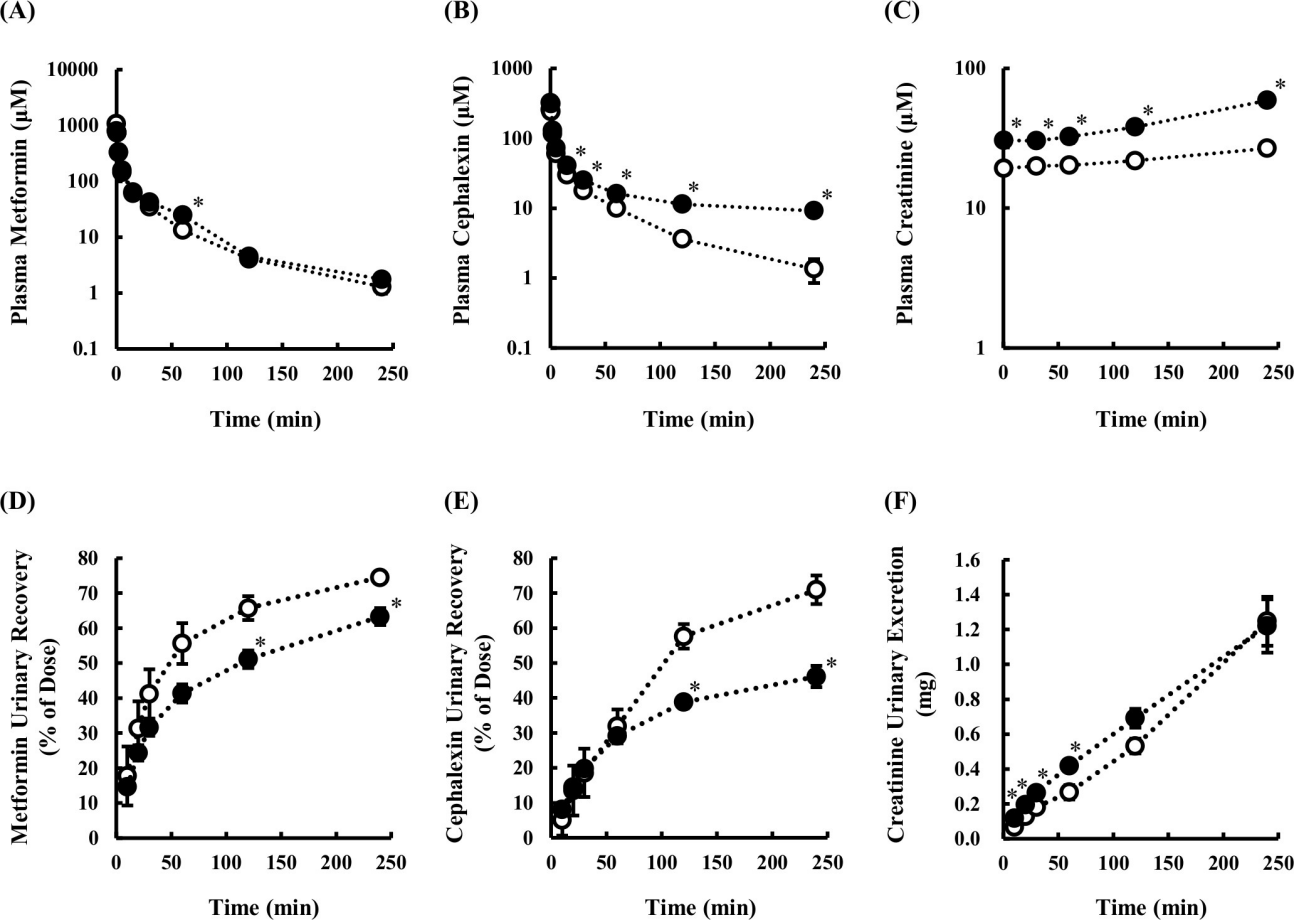
**Plasma concentration-time profiles of metformin (A), cephalexin (B) and creatinine (C) and cumulative urinary excretions of metformin (D), cephalexin (E) and creatinine (F) in control (open circles) and hyperuricemic rats (closed circles).** Concentrations of metformin, cephalexin and creatinine in plasma and urine were measured up to 4 h after intravenous administration of metformin (30 mg/kg) or cephalexin (10 mg/kg). Creatinine concentration represents endogenous creatinine. Data are presented as mean ± SEM (n = 3). *p < 0.05 vs. control rats.

Metformin, cephalexin and creatinine were selected for the pharmacokinetic study, since they are excreted intact in urine and are all substrates of Mate1 [22]. Cephalexin is also a substrate of Oat1 [23, 24], and metformin and creatinine are also substrates of Oct2 [25, 26]. Plasma concentration-time curves and urinary excretion profiles of cephalexin and metformin after intravenous administration to control and hyperuricemic rats on Day 10 are shown in Fig 3, and the pharmacokinetic parameters are summarized in Table 2.

**Table 2:** Pharmacokinetic parameters of metformin, cephalexin and creatinine in control and hyperuricemic rats after intravenous administration of metformin or cephalexin.

Compounds	Rat	AUC_0-4_			AUC_inf_			CL_R_			fu	(CL_R_/f_u_)/CL_Inulin_			Urinary Recovery			K_p, kidney_		
		μM·min			μM·min			mL/min/kg							% of Dose/4 h					
Metformin	Control	5530	±	198	5638	±	173	23.0	±	0.6	0.85 [27]	4.03	±	0.11	74.5	±	1.3	16.6	±	9.9
(30 mg/kg)	Hyperuricemia	6100	±	648	6223	±	641	18.3	±	2.8	0.85 [27]	4.01	±	0.62	63.3	±	2.4*	90.3	±	19.8*
	Fold Change	1.10			1.10			0.80			NA	1.00			0.85			5.44		
Cephalexin	Control	2629	±	221*	2770	±	274	7.96	±	1.15	0.82 [28]	1.44	±	0.21	71.0	±	4.1	9.76	±	1.32
(10 mg/kg)	Hyperuricemia	4625	±	28*	7381	±	258*	2.87	±	0.20*	0.82 [28]	0.65	±	0.05*	46.1	±	3.1*	15.1	±	0.4*
	Fold Change	1.76			2.66			0.36			NA	0.45			0.65			1.55		
Creatinine	Control	5373	±	62	NC			10.9	±	1.2	1.0	1.62	±	0.17	NA			10.2	±	0.7
	Hyperuricemia	9807	±	590*	NC			5.84	±	0.21*	1.0	1.09	±	0.04*	NA			13.7	±	1.8
	Fold Change	1.83			NC			0.54			NA	0.67			NA			1.34		

Values were determined from the data shown in Fig 3. Values are presented as mean ± SEM (n = 3). f_u_ of creatinine was set at 1.0.

*p < 0.05 vs. control rats.

NA, not applicable; NC, not calculated.

Although the plasma concentration and renal clearance of metformin after intravenous administration at 30 mg/kg were not affected by hyperuricemia, kidney accumulation (K_p, kidney_) was significantly increased, and urinary recovery up to 4 h was slightly but significantly decreased.

**Fig 4:**
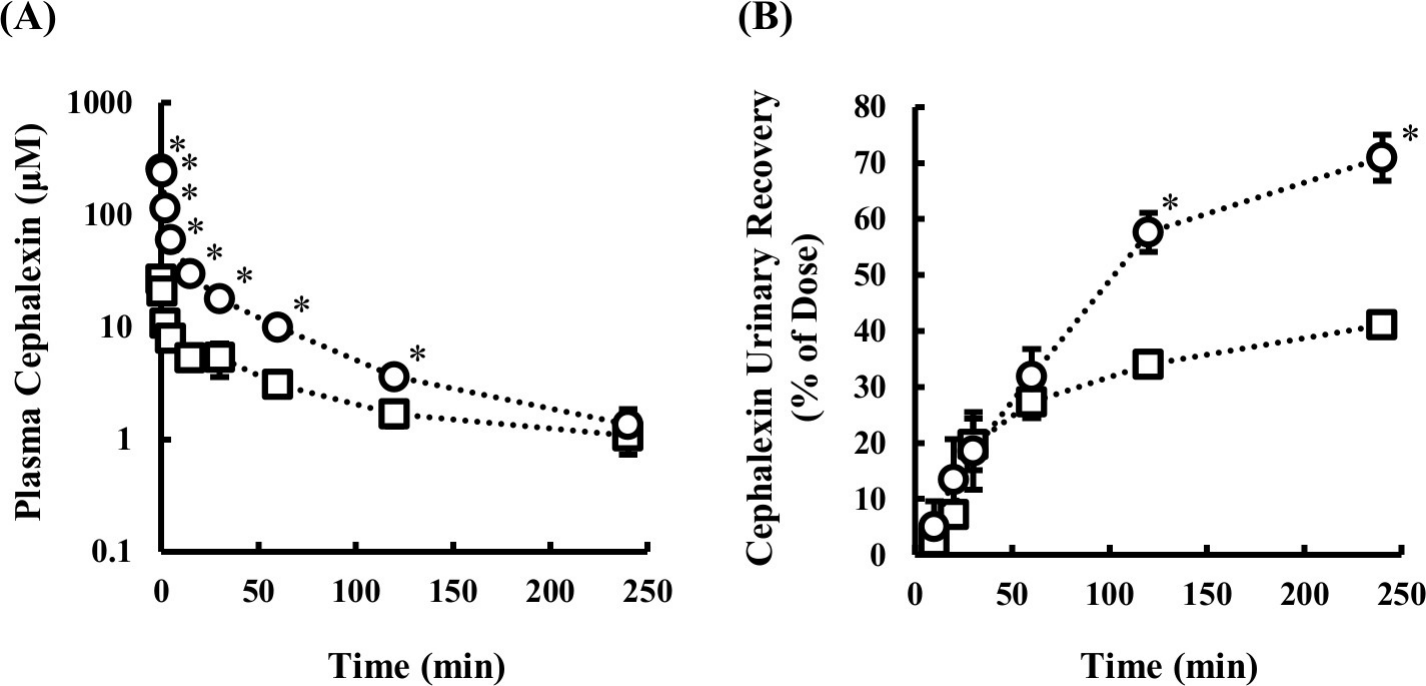
Plasma concentration-time profile (A) and cumulative urinary excretion (B) of cephalexin after administration of high (10 mg/kg, circles) and low (1 mg/kg, squares) doses. Concentrations of cephalexin in plasma and urine were measured up to 4 h after intravenous administration of cephalexin. Data are presented as mean ± SEM (n = 3). *p < 0.05 vs. 1 mg/kg dose group.

After intravenous administration of 10 mg/kg cephalexin, the plasma concentration and kidney accumulation were higher in hyperuricemic rats than in control rats. The ratio of renal clearance of cephalexin ((CL_R_/f_u_)/CL_Inulin_) to that of inulin was 1.44 in control rats, indicating that tubular secretion is the dominant pathway of cephalexin clearance. However, the clearance ratio in hyperuricemic rats was decreased to 0.65, which suggests that extensive reabsorption occurs. Next, we compared the pharmacokinetic parameters of cephalexin in control rats after intravenous administration of a low dose (1 mg/kg) with that at the dose of 10 mg/kg. The plasma concentration- and cumulative urinary excretion-time curves and pharmacokinetic parameters are shown in Fig 4 and Table 3. The plasma concentration was increased at the higher dose, while the urinary recovery was decreased. Estimated total clearance and renal clearance were much higher in rats administered 10 mg/kg (10.6 mL/min/kg and 7.96 mL/min/kg, respectively) than in rats administered 1 mg/kg (3.57 mL/min/kg and 1.88 mL/min/kg, respectively). The ratio of renal clearance of cephalexin to inulin clearance was remarkably different at the doses of 1 and 10 mg/kg, being 0.34 and 1.44, respectively. Accordingly, reabsorption is considered to be dominant at the low dose of cephalexin, whereas secretion is dominant at the high dose. Thus, renal disposition of cephalexin in hyperuricemic rats appears to be similar to that in control rats given the low dose of cephalexin.

**Table 3:** Pharmacokinetic parameters of cephalexin in control rats after intravenous administration of cephalexin.

Dose	AUC_0-4_			AUC_inf_			CL_tot_			CL_R_			CL_cr_			fu	(CL_R_/f_u_)/CL_Inulin_			Urinary Recovery		
	μM·min			μM·min			mL/min/kg			mL/min/kg			mL/min/kg							% of dose/4 h		
1 mg/kg	656	±	79	901	±	209	3.57	±	0.84	1.88	±	0.31	7.66	±	0.53	0.82 [28]	0.34	±	0.06	41.1	±	1.5
10 mg/kg	2629	±	221*	2770	±	274*	10.6	±	1.2*	7.96	±	1.15*	10.9	±	1.2	0.82 [28]	1.44	±	0.21*	71.0	±	4.1*

Values were determined from the data shown in Fig 4. Values are presented as mean ± SEM (n = 3).

*p < 0.05 vs. 1 mg/kg dose group.

As regards creatinine, the plasma concentration was increased, and renal clearance was decreased in hyperuricemic rats (Table 2). Creatinine accumulation in kidney tended to be increased in hyperuricemic rats, though this was not statistically significant. The ratio of renal clearance of creatinine to that of inulin was decreased in hyperuricemic rats, but the levels were higher than unity in both control and hyperuricemic rats, suggesting that secretion is dominant.

### Protein expression of Mate1 in kidney

**Fig 5:**
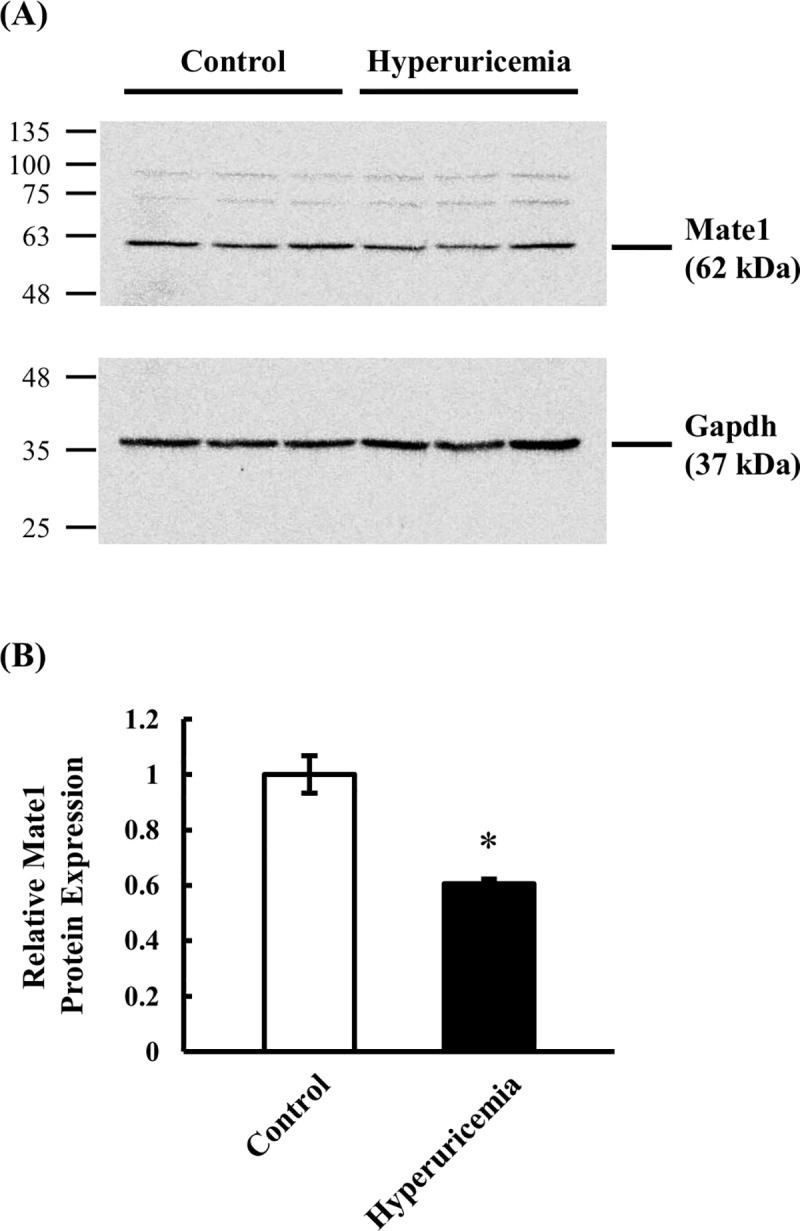
Protein expression level of kidney Mate1 in control and hyperuricemic rats. Western blot analysis was conducted using whole kidney lysate. (A) Typical result detected with anti-Mate1 and anti-Gapdh antibodies on the same membrane is shown. (B) Relative Mate1 density normalized to Gapdh density in hyperuricemic rats (closed column) are shown as the ratio to those in control rats (open column). Data are presented as mean ± SEM (n = 3). *p < 0.05 vs. control rats.

Since pharmacokinetics of Mate1 substrates, metformin, cephalexin and creatinine, in hyperuricemic rats were changed compared to control rats, a protein expression of Mate1 in kidney was assessed by Western blot analysis. A reduction of the protein expression of kidney Mate1 was observed in whole kidney lysates. The Mate1 protein expression was significantly decreased to 60.7% in hyperuricemic rats, compared to control rats (Fig 5).

### Effects of oxonic acid, adenine and uric acid on MATE1-mediated uptake

**Fig 6:**
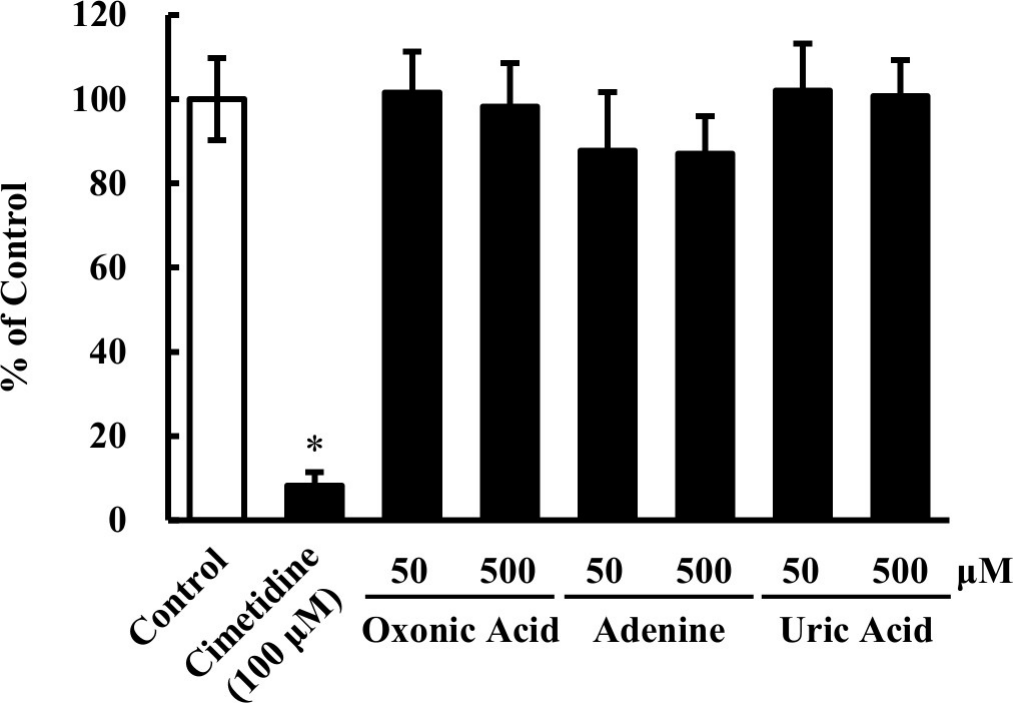
Effects of oxonic acid, adenine and uric acid on uptake of MPP^+^ by MATE1-expressing cells. Uptake of [^3^H]MPP^+^ (2.5 nM) by HEK293/MATE1 and HEK293/mock cells was performed at 37°C for 0.5 min in the absence or presence of oxonic acid, adenine or uric acid. Cimetidine was used as a positive control of Mate1 inhibitor. Data are presented as mean ± SEM (n = 3). *p < 0.05 vs. control rats (Dunnett’s test).

Inhibitory effects of oxonic acid, adenine and uric acid on Mate1 activity were investigated, and the results are depicted in Fig 6. As the Mate1 activity, uptake of [^3^H]MPP^+^ was conducted using human MATE1-expressing HEK293 cells and the mock cells. The percentages of control values were 101.5% and 98.2% for oxonic acid, 87.7% and 87.0% for adenine, 102.0% and 100.7% for uric acid at 50 and 500 μM, respectively. No inhibitory effects of oxonic acid, adenine and uric acid (50 and 500 μM of each) were observed on MATE1-mediated MPP^+^ uptake, while Mate1 inhibitor cimetidine decreased its uptake to 8.2%.

## Discussion

There are several reports that clinically used drugs affect serum uric acid level through interaction with transporters or enzymes related to the disposition of uric acid. Since some uric acid transporters are also involved in drug disposition, it is not surprising that hyperuricemia also affects drug transporters [11, 14, 29]. Here, we examined the effect of hyperuricemia on drug disposition, focusing on renal organic cation transporters whose expression level is significantly reduced in hyperuricemic rats (Fig 2).

First of all, we established hyperuricemic rats by short-term (10 days) co-administration of oxonic acid and adenine as a chronic hyperuricemia model, by a modification of previously reported method [30]. Xanthine oxidase inhibitor oxonic acid alone increases the plasma uric acid concentration in rats by inhibiting further metabolism of uric acid to allantoin, but the increase is not high enough in our preliminary study. On the other hand, adenine administration for 4 to 16 weeks has been used to establish a chronic kidney failure model exhibiting increased plasma uric acid concentration, as well as increased serum creatinine level and BUN, and decreased glomerular filtration rate (GFR) [31, 32]. The combined treatment for a short period (10 days) enabled us to prepare hyperuricemic rats with significantly increased plasma uric acid concentration of up to about 6 mg/dL (Fig 1), but without marked signs of chronic kidney failure (Table 1). The present model showed only mild changes in markers of kidney functions, such as BUN (15.4 to 34.2 mg/dL, Table 1) and inulin clearance (GFR, 6.72 to 5.36 mL/min/kg), compared with those observed in rats fed adenine alone for 4 weeks (BUN: 6.96 to 82.3 mg/dL, GFR: 2.59 to 0.10 mL/min) [32]. The reason for this is presumably that the severity of kidney failure in adenine-fed animals depends on the dose and duration of adenine administration [18]. Accordingly, a short-term adenine treatment with co-administration of oxonic acid should be suitable to examine the effect of hyperuricemia on transporter expression with a minimal complication of kidney failure. Although a role of factors other than elevated uric acid cannot be ruled out, these rats appear to be a good model of human hyperuricemia, in which variable changes in physiological measures are observed.

Expression of multiple kidney transporters was suppressed in our hyperuricemia model (Fig 2). It has already been reported that expression of Oct2 and Oat1, which is present at the basolateral membrane of tubular epithelial cells, is decreased in a hyperuricenic model obtained by 10-day feeding of oxonic acid and uric acid in rats [16]. In the present study, we focused especially on Mate1, an organic cation transporter at the renal tubular apical membrane. It has already been reported that Mate1 expression is decreased in a rat kidney failure model generated by adenine administration for 4 weeks [32]. In the present model, in contrast, renal failure is mild at most, as indicated by the insignificant change of inulin clearance and minimal increase of BUN (Table 1). Therefore the present model should be more suitable to examine the specific effects of hyperuricemia.

Notably, in the hyperuricemic rats, the plasma concentration and renal clearance of creatinine, a clinically used biomarker of kidney function, were significantly increased and decreased, respectively (Fig 3C and 3F). In addition, the renal clearance ratio of creatinine to inulin was decreased from 1.62 to 1.09 in the hyperuricemic rats (Table 2). This observation could be explained in terms of decreased tubular secretion via Oct2 and/or Mate1 transporters, but not by a decrease of GFR, since the data were corrected for inulin clearance. Habu Y *et al*., reported that a decrease in Oct2 protein expression in hyperurecemic rats led to a decrease in the accumulation of Oct2 substrates, such as tetraethylammonium and cimetidine, into kidney slices [16]. According to this report, the lowered Oct2 mRNA levels decreased creatinine uptake from the blood into the tubular cells. This is a possible mechanism of the decrease in renal Oct2-mediated creatinine clearance observed in hyperuricemic rats. On the other hand, current study demonstrated that the decrease in Mate1 protein expression probably led to the decrease in creatinine excretion from the tubular cells into lumen. The accumulation of creatinine in kidney could be due to the decrease in Mate1 protein expression. From these results, it is strongly suggests that the decrease in Mate1 expression is one of the major causes of the decrease in renal creatinine clearance observed in hyperuricemic rats, in addition to the decrease in Oct2 expression.

Metformin is secreted into the urine via Oct2 and Mate1 in the same manner as creatinine [22, 25]. Plasma concentration and renal clearance of metformin in the hyperuricemic rats showed no statistically significant change (Fig 3A and 3D). On the other hand, accumulation of metformin in the kidney tissue (K_p, kidney_) increased significantly from 16.6 to 90.3 (Table 2). The renal clearance of metformin is considered to be a blood flow rate-limited in our study (rat blood flow 23 mL/min/kg [33]) as well as reported in mice [34], which indicates that the contribution of Oct2 to metformin elimination from the blood was negligible. Since Mate1 is a key transporter which excretes metformin from the tubular cells into the lumen, it is reasonable that decreased Mate1 expression would affect kidney tissue accumulation, but not the apparent plasma profile of metformin in rats. As metformin and creatinine are substrates of Oct1/2 as well as Mate1 in kidney, the elevated endogenous creatinine may affect pharmacokinetics of metformin. However, the renal clearance of metformin is considered to be a blood flow rate-limited, which means apparently less affected by transporter-mediated interaction. Therefore, the renal clearance of metformin is considered to be unlikely affected by creatinine via competition on Oct1/2 transporters in kidney.

Cephalexin is taken up into kidney tissues via transporters such as Oat1 and secreted via Mate1 [23, 24, 35]. In addition, it is reabsorbed via peptide transporters Pept1 and Pept2 (Slc15a2) [36–38]. The observed changes in plasma concentration, kidney tissue accumulation and renal clearance of cephalexin could be well explained by the changes of these transporters. Urinary recovery of cephalexin was significantly delayed or decreased in the hyperuricemic rats (Fig 3E). Since cephalexin is reabsorbed via Pept transporters, it was considered that the reabsorption efficiency is increased due to the decreased tubular intra-lumen concentration of cephalexin. To confirm this, we evaluated the dose dependence of cephalexin disposition by decreasing the dose to 1 mg/kg from 10 mg/kg in control rats (Fig 4). As expected, the renal clearance of cephalexin was greatly decreased at the low dose (1 mg/kg), and the clearance ratio was less than unity (0.34), whereas it was higher than unity (1.44) at the high dose (10 mg/kg, Table 3). The findings that reabsorption is predominant at the low dose, but is not efficient at the high dose, can be explained in terms of saturation of Pept-mediated reabsorption of cephalexin. Therefore, the decrease of renal clearance ratio to less than unity in hyperuricemia is considered to be due to more efficient reabsorption as a consequence of the decreased tubular concentration of cephalexin resulting from the lower Mate1 expression. When the same mechanism is assumed, the clearance ratio probably remains to be less than 1, along with a decrease in renal clearance and an increase in K_p,kidney_ through decreasing Oat1 and Mate1 activities in hyperuricemic rats at 1 mg/kg. This mechanism may account predominantly for the substantial change in renal handling of cephalexin in hyperuricemic rats, even though the Pept1 and Pept2 mRNA levels in the hyperuricemic rats were lower than in control rats (Fig 2).

**Fig 7:**
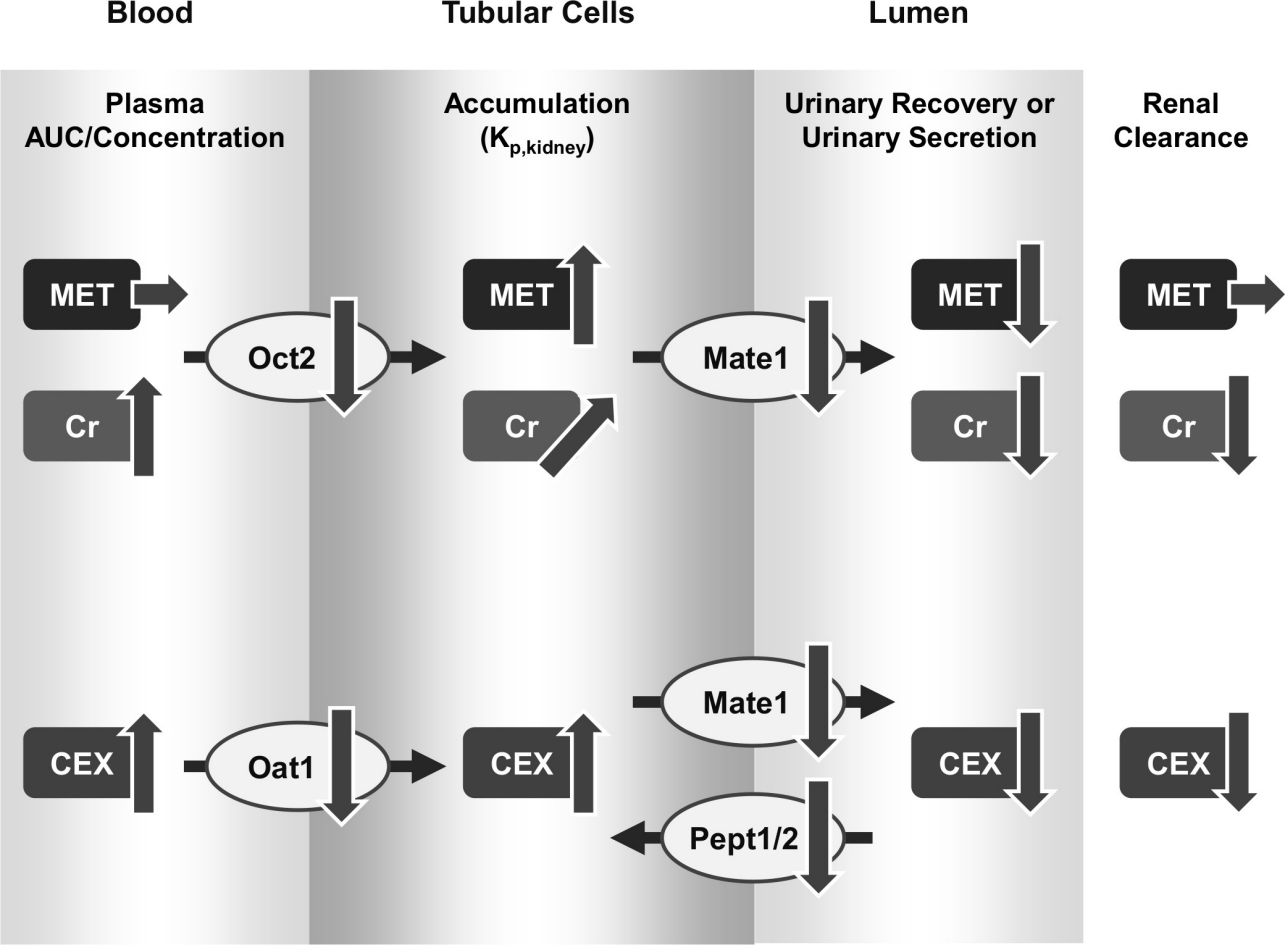
Schematic figure recapitulating the effects of hyperuricemia on mRNA and protein expressions of transporters in kidney, as well as its putative consequences regarding to the pharmacokinetics. Changes in plasma AUC and concentration of each drug are shown in the blood column. Changes in kidney accumulation (K_p,kidney_) of each drug are shown in the tubular cells column. Changes in urinary secretion of each drug are shown in lumen column. In regard to transporters, changes in mRNA and/or protein expression are shown. Arrows show changes as follows: ↑, increase; ↓, decrease; →, no change. Metformin and creatinine are substrates of Oct2 and Mate1. Cephalexin is a substrate of Oat2, Mate1 and Pept1/2. MET, metformin; Cr, creatinine; CEX, cephalexin.

Since Mate1 is a key transporter to explain pharmacokinetic alterations of tested drugs by hyperuricemia, the protein level of kidney Mate1 in hyperuricemia was measured, and the significant decrease to 60.5% of normal rats was observed (Fig 5), which is comparable with the change in mRNA expression (Fig 2). This change in protein expression caused an increase in K_p,kidney_ of metformin, cephalexin and creatinine. This observation confirmed that pharmacokinetic changes of metformin, cephalexin and creatinine are associated with a decrease of Mate1 expression. As other factors affecting Mate1 activity, changes in Mate1 distribution to the BBM and H^+^ gradient (*i*.*e*. driving force) are considered. Further studies are needed in order to clarify exact mechanisms affecting pharmacokinetics of these drugs. The effects of hyperuricemia on mRNA and protein expressions of transporters in kidney, as well as its putative consequences regarding to the pharmacokinetics is summarized in Fig 7.

Zhang G. *et al*., recently reported that expression of kidney Oct1, Oct2 and Mate1 and hepatic Mate1 is increased in a hyperuricenic model, which is prepared by a single intraperitoneal injection of oxonic acid in rats [39]. This model seems to be an acute hyperuricemic model. On the other hand, Habu Y. *et al*., reported that mRNA and protein expressions of Oct2 and Oat1 in kidney are decreased in a hyperuricenic model, which is prepared by 10-day feeding of oxonic acid and uric acid in rats [16]. In the present study, a 10-day administration of oxonic acid and adenine to rats resulted in a decrease in mRNA and/or protein expression of Oct2, Oat1 and Mate1. Both models, Habu Y. *et al*. and the present study, are considered as a chronic hyperuricemic model. A main difference is that the chronic model is exposed to elevated serum uric acid for a longer time than the acute model. Therefore, the discrepancy in the effect of hyperuricemia on transporter expression may be due to the exposure period to elevated serum uric acids; however, further studies are needed.

Administered compounds, oxonic acid and adenine, and elevated uric acid may potentially inhibit Mate1 activity; however, they have no inhibitory effects on human MATE1-mediated transport up to 500 μM (87.0 to 102.0% of control, Fig 6). Plasma uric acid levels on Day 10 were 2.38 to 4.73 mg/dL (*i*.*e*. 142 to 281 μM). The plasma concentration of oxonic acid was lower than the detection limit (100 μM) in the current analytical method. The plasma concentration of adenine was lower than 100 μM. Therefore, competitive inhibition of Mate1 by these compounds was unlikely a cause of pharmacokinetic changes of metformin, cephalexin, and creatinine.

In conclusion, we observed similar but distinct changes in the renal dispositions of creatinine, metformin and cephalexin in our hyperuricemic rat model, which exhibited only minimal signs of kidney failure. Although this hyperuricemia model may be influenced by changes other than hyperuricemia, our results are consistent with the idea that high levels of uric acid in the plasma can alter drug disposition by inducing changes of transporter expression in the kidney. In particular, our results indicate that decreased Mate1-mediated apical membrane transport plays a major role in the altered pharmacokinetics of all three drugs in hyperuricemic animals.

## Supporting information

S1 Table Primers for quantitative PCR. (DOCX)

S2 Table Plasma concentration of uric acid in control rats and hyperuricemic rats during the 10-day administration period (dataset of Fig 1). (DOCX)

S3 Table Plasma concentrations of BUN and creatinine and inulin clearance in control and hyperuricemic rats (dataset of Table 1). (DOCX)

S4 Table Relative mRNA expression levels of transporters in kidney of control and hyperuricemic rats (dataset of Fig 2). (DOCX)

S5 Table Plasma concentrations of metformin, cephalexin and creatinine and cumulative urinary excretions of metformin, cephalexin and creatinine in control and hyperuricemic rats (dataset of Fig 3). (DOCX)

S6 Table Pharmacokinetic parameters of metformin, cephalexin and creatinine in control and hyperuricemic rats after intravenous administration of metformin or cephalexin (dataset of Table 2). (DOCX)

S7 Table Plasma concentration and cumulative urinary excretion of cephalexin after administration of high (10 mg/kg) and low (1 mg/kg) doses (dataset of Fig 4). (DOCX)

S8 Table Pharmacokinetic parameters of cephalexin in control rats after intravenous administration of cephalexin (dataset of Table 3). (DOCX)

S9 Table Protein expression level of kidney Mate1 in control and hyperuricemic rats (dataset of Fig 5). (DOCX)

S10 Table Effects of oxonic acid, adenine and uric acid on uptake of MPP^+^ by MATE1-expressing cells (dataset of Fig 6). (DOCX)
