# Supplementary material for: Changes of drug pharmacokinetics mediated by downregulation of kidney organic cation transporters Mate1 and Oct2 in a rat model of hyperuricemia
Source: PLoS One. 2019 Apr 5;14(4):e0214862. doi: 10.1371/journal.pone.0214862 (PMC6450621; doi:10.1371/journal.pone.0214862)
Supplement: S1 Table — (DOCX) [file pone.0214862.s001.docx]

**S1 Table. Primers for quantitative PCR**

| Gene name | Forward (5’ to 3’) | Reverse (5’ to 3’) |
| --- | --- | --- |
| Gapdh | GGGCTCTCTGCTCCTC | AGGCGTCCGATACGGC |
| Mate1 (Slc47a1) | CTCTCAGGAGCCTGCTCACCC | GACAGGCAAAGCTTGTTGCTGG |
| Oat1 (Slc22a6) | TCAGCAAAGATGGAGGTCTGG | TAAAGCGGAGGCAAGATTCG |
| Oat3 (Slc22a8) | CTGAAGGAGATGGCCCAGTC | CCAGGTCAGGATAGGCTTGC |
| Oct1 (Slc22a1) | TTTAACCTGGTGTGTGGAGACG | AGGAAGAAGCCCAAGTTCACAC |
| Oct2 (Slc22a2) | CGGTGCTATGATGATTGGCTAC | CCAGGCATAGTTGGGAGAAATC |
| Mrp2 (Abcc2) | TTTTGACACAACTCCCACAGG | CAGCGATGCCAAAGAAACAC |
| Mrp4 (Abcc4) | CAGGGCTGCTGAATGCAATA | TTGGATTCGGGAAGACTGAGA |
| Urat1 (Slc22a12) | TGAGGATGGCTGGGTTTACG | CCAGCCAGGAAGATGGACTG |
| Glut9 (Slc2a9) | GCTTGCCCTAGCTTCCCTGA | AGGAAGGAGGACCCGAAGG |
| Pept1 (Slc15a1) | GTGTGGGGCCCCAATCTATACCGT | GTTTGTCTGTGAGACAGGTTCCAA |
| Pept2 (Slc15a2) | CCTCCAAAGAAGTCACCTCCG | CACATCTCCTCTCAGCATGGG |
| Mdr1a (Abcb1a) | GGAGGCTTGCAACCAGCATTC | CTGTTCTGCCGCTGGATTTC |
| Mdr1b (Abcb1b) | CTGCTATCATCCACGGAACC | GCTGACGGTCTGTGTACTGTT |
| Bcrp (Abcg2) | CCGGAAAACAGCTGAGAAAG | GAAATTGGCAGGTTGAGGTG |
