# Supplementary material for: Changes of drug pharmacokinetics mediated by downregulation of kidney organic cation transporters Mate1 and Oct2 in a rat model of hyperuricemia
Source: PLoS One. 2019 Apr 5;14(4):e0214862. doi: 10.1371/journal.pone.0214862 (PMC6450621; doi:10.1371/journal.pone.0214862)
Supplement: S2 Table — (DOCX) [file pone.0214862.s002.docx]

**S2 Table. Plasma concentration of uric acid in control rats and hyperuricemic rats during the 10-day administration period (dataset of Fig 1).**

|  |  | Plasma uric acid concentration (mg/dL) | | | | |
| --- | --- | --- | --- | --- | --- | --- |
|  |  | Day 0 | Day 1 | Day 3 | Day 7 | Day 10 |
| Control rats |  | 0.68 | 0.32 | 0.22 | 0.25 | 0.38 |
|  |  | 0.61 | 0.26 | 0.20 | 0.36 | 0.49 |
|  |  | 0.66 | 0.25 | 0.18 | 0.23 | 0.28 |
|  | Mean | 0.65 | 0.28 | 0.20 | 0.28 | 0.38 |
|  | SEM | 0.02 | 0.02 | 0.01 | 0.04 | 0.06 |
| Hyperuricemic rats |  | 0.36 | 3.13 | 3.18 | 3.19 | 4.73 |
|  |  | 0.39 | 3.15 | 2.89 | 3.93 | 3.05 |
|  |  | 0.43 | 3.34 | 3.60 | 3.11 | 3.45 |
|  |  | 1.33 | 2.48 | 11.47 | 4.89 | 3.20 |
|  |  | 0.96 | 2.71 | 6.74 | 8.51 | 2.38 |
|  |  | 0.58 | 1.31 | 6.22 | 3.07 | 3.84 |
|  |  | 0.59 | 2.44 | 8.21 | 1.14 | 2.71 |
|  | Mean | 0.66 | 2.65 | 6.05 | 3.98 | 3.34 |
|  | SEM | 0.13 | 0.26 | 1.18 | 0.87 | 0.29 |
|  | p value | 0.96 | 0.0004 | 0.01 | 0.03 | 0.0002 |

Unpaired Student’s t-test was used to analyze differences between groups.
