# Supplementary material for: Changes of drug pharmacokinetics mediated by downregulation of kidney organic cation transporters Mate1 and Oct2 in a rat model of hyperuricemia
Source: PLoS One. 2019 Apr 5;14(4):e0214862. doi: 10.1371/journal.pone.0214862 (PMC6450621; doi:10.1371/journal.pone.0214862)
Supplement: S3 Table — (DOCX) [file pone.0214862.s003.docx]

**S3 Table. Plasma concentrations of BUN and creatinine and inulin clearance in control and hyperuricemic rats (dataset of Table 1).**

**(A) BUN**

|  | Control rats | Hyperuricemic rats |
| --- | --- | --- |
| BUN | 13.1 | 31.4 |
| (mg/dL) | 13.8 | 31.8 |
|  | 16.4 | 42.8 |
|  | 18.4 | 33.7 |
|  | - | 31.8 |
|  | - | 33.4 |
|  | - | 34.6 |
| Mean | 15.4 | 34.2 |
| SEM | 1.2 | 1.5 |
| p value | - | 0.00001 |

Unpaired Student’s t-test was used to analyze differences between groups.

**(B) Plasma creatinine**

|  | Control rats | Hyperuricemic rats |
| --- | --- | --- |
| Plasma | 13.0 | 49.9 |
| Creatinine | 12.5 | 42.8 |
| (µM) | 12.8 | 29.5 |
|  | - | 18.0 |
|  | - | 31.8 |
|  | - | 29.9 |
|  | - | 32.9 |
| Mean | 12.8 | 33.6 |
| SEM | 0.1 | 3.9 |
| p value | - | 0.009 |

Unpaired Student’s t-test was used to analyze differences between groups.

**(C) Inulin clearance**

|  | Control rats | Hyperuricemic rats |
| --- | --- | --- |
| Inulin | 5.90 | 7.92 |
| Clearance | 7.51 | 4.99 |
| (mL/min/kg) | 6.75 | 6.15 |
|  | - | 4.14 |
|  | - | 3.58 |
| Mean | 6.72 | 5.36 |
| SEM | 0.46 | 0.77 |
| p value | - | 0.26 |

Unpaired Student’s t-test was used to analyze differences between groups.
