# Supplementary material for: Changes of drug pharmacokinetics mediated by downregulation of kidney organic cation transporters Mate1 and Oct2 in a rat model of hyperuricemia
Source: PLoS One. 2019 Apr 5;14(4):e0214862. doi: 10.1371/journal.pone.0214862 (PMC6450621; doi:10.1371/journal.pone.0214862)
Supplement: S4 Table — (DOCX) [file pone.0214862.s004.docx]

**S4 Table. Relative mRNA expression levels of transporters in kidney of control and hyperuricemic rats (dataset of Fig 2).**

**(A) Mate1**

|  | Ct  (Mate1) | Ct  (Gapdh) | Relative level | Mean | SE | p value |
| --- | --- | --- | --- | --- | --- | --- |
| Control rats | 23.53 | 16.23 | 0.82 | 1.00 | 0.10 |  |
|  | 23.10 | 16.29 | 1.16 |  |  |  |
|  | 22.90 | 15.91 | 1.02 |  |  |  |
| Hyperuricemic rats | 26.20 | 18.03 | 0.45 | 0.54 | 0.03 | 0.0008 |
|  | 25.28 | 17.62 | 0.64 |  |  |  |
|  | 26.23 | 18.32 | 0.54 |  |  |  |
|  | 26.80 | 18.61 | 0.44 |  |  |  |
|  | 23.59 | 15.73 | 0.56 |  |  |  |
|  | 24.26 | 16.58 | 0.63 |  |  |  |

Unpaired Student’s t-test was used to analyze differences between groups.

**(B) Oat1**

|  | Ct  (Oat1) | Ct  (Gapdh) | Relative level | Mean | SE | p value |
| --- | --- | --- | --- | --- | --- | --- |
| Control rats | 20.23 | 16.23 | 0.77 | 1.00 | 0.13 |  |
|  | 19.89 | 16.29 | 1.01 |  |  |  |
|  | 19.24 | 15.91 | 1.22 |  |  |  |
| Hyperuricemic rats | 22.97 | 18.03 | 0.40 | 0.43 | 0.04 | 0.001 |
|  | 22.08 | 17.62 | 0.56 |  |  |  |
|  | 23.09 | 18.32 | 0.45 |  |  |  |
|  | 24.14 | 18.61 | 0.27 |  |  |  |
|  | 20.72 | 15.73 | 0.39 |  |  |  |
|  | 21.13 | 16.58 | 0.52 |  |  |  |

Unpaired Student’s t-test was used to analyze differences between groups.

**(C) Oat3**

|  | Ct  (Oat3) | Ct  (Gapdh) | Relative level | Mean | SE | p value |
| --- | --- | --- | --- | --- | --- | --- |
| Control rats | 22.69 | 17.40 | 0.62 | 1.00 | 0.19 |  |
|  | 21.12 | 16.81 | 1.22 |  |  |  |
|  | 20.46 | 16.09 | 1.17 |  |  |  |
| Hyperuricemic rats | 22.73 | 16.10 | 0.24 | 0.58 | 0.13 | 0.13 |
|  | 22.36 | 16.40 | 0.39 |  |  |  |
|  | 21.08 | 16.04 | 0.73 |  |  |  |
|  | 20.80 | 16.46 | 1.19 |  |  |  |
|  | 22.20 | 16.08 | 0.35 |  |  |  |
|  | 21.89 | 16.55 | 0.60 |  |  |  |

Unpaired Student’s t-test was used to analyze differences between groups.

**(D) Oct1**

|  | Ct  (Oct1) | Ct  (Gapdh) | Relative level | Mean | SE | p value |
| --- | --- | --- | --- | --- | --- | --- |
| Control rats | 22.41 | 16.23 | 0.71 | 1.00 | 0.18 |  |
|  | 22.04 | 16.29 | 0.96 |  |  |  |
|  | 21.20 | 15.91 | 1.32 |  |  |  |
| Hyperuricemic rats | 24.60 | 18.03 | 0.55 | 0.59 | 0.09 | 0.06 |
|  | 23.63 | 17.62 | 0.80 |  |  |  |
|  | 25.00 | 18.32 | 0.51 |  |  |  |
|  | 26.44 | 18.61 | 0.23 |  |  |  |
|  | 22.37 | 15.73 | 0.52 |  |  |  |
|  | 22.40 | 16.58 | 0.92 |  |  |  |

Unpaired Student’s t-test was used to analyze differences between groups.

**(E) Oct2**

|  | Ct  (Oct2) | Ct  (Gapdh) | Relative level | Mean | SE | p value |
| --- | --- | --- | --- | --- | --- | --- |
| Control rats | 18.41 | 16.23 | 0.90 | 1.00 | 0.06 |  |
|  | 18.19 | 16.29 | 1.09 |  |  |  |
|  | 17.91 | 15.91 | 1.02 |  |  |  |
| Hyperuricemic rats | 21.19 | 18.03 | 0.45 | 0.58 | 0.10 | 0.03 |
|  | 20.45 | 17.62 | 0.57 |  |  |  |
|  | 21.36 | 18.32 | 0.49 |  |  |  |
|  | 20.52 | 18.61 | 1.08 |  |  |  |
|  | 18.63 | 15.73 | 0.54 |  |  |  |
|  | 20.08 | 16.58 | 0.36 |  |  |  |

Unpaired Student’s t-test was used to analyze differences between groups.

**(F) Mrp2**

|  | Ct  (Mrp2) | Ct  (Gapdh) | Relative level | Mean | SE | p value |
| --- | --- | --- | --- | --- | --- | --- |
| Control rats | 21.89 | 17.40 | 2.23 | 1.00 | 0.62 |  |
|  | 24.26 | 16.81 | 0.29 |  |  |  |
|  | 22.79 | 16.09 | 0.48 |  |  |  |
| Hyperuricemic rats | 23.00 | 16.10 | 0.42 | 0.47 | 0.02 | 0.24 |
|  | 23.05 | 16.40 | 0.50 |  |  |  |
|  | 22.51 | 16.04 | 0.57 |  |  |  |
|  | 23.04 | 16.46 | 0.52 |  |  |  |
|  | 22.98 | 16.08 | 0.42 |  |  |  |
|  | 23.46 | 16.55 | 0.42 |  |  |  |

Unpaired Student’s t-test was used to analyze differences between groups.

**(G) Mrp4**

|  | Ct  (Mrp4) | Ct  (Gapdh) | Relative level | Mean | SE | p value |
| --- | --- | --- | --- | --- | --- | --- |
| Control rats | 22.26 | 17.40 | 1.06 | 1.00 | 0.11 |  |
|  | 21.55 | 16.81 | 1.15 |  |  |  |
|  | 21.39 | 16.09 | 0.78 |  |  |  |
| Hyperuricemic rats | 21.06 | 16.10 | 0.99 | 1.17 | 0.06 | 0.21 |
|  | 20.88 | 16.40 | 1.38 |  |  |  |
|  | 20.88 | 16.04 | 1.08 |  |  |  |
|  | 20.98 | 16.46 | 1.34 |  |  |  |
|  | 20.73 | 16.08 | 1.23 |  |  |  |
|  | 21.48 | 16.55 | 1.01 |  |  |  |

Unpaired Student’s t-test was used to analyze differences between groups.

**(H) Urat1**

|  | Ct  (Urat1) | Ct  (Gapdh) | Relative level | Mean | SE | p value |
| --- | --- | --- | --- | --- | --- | --- |
| Control rats | 22.84 | 17.40 | 0.83 | 1.00 | 0.09 |  |
|  | 21.94 | 16.81 | 1.03 |  |  |  |
|  | 21.07 | 16.09 | 1.14 |  |  |  |
| Hyperuricemic rats | 22.67 | 16.10 | 0.38 | 0.51 | 0.05 | 0.002 |
|  | 22.97 | 16.40 | 0.38 |  |  |  |
|  | 22.25 | 16.04 | 0.49 |  |  |  |
|  | 22.06 | 16.46 | 0.74 |  |  |  |
|  | 22.06 | 16.08 | 0.57 |  |  |  |
|  | 22.72 | 16.55 | 0.50 |  |  |  |

Unpaired Student’s t-test was used to analyze differences between groups.

**(I) Glut9**

|  | Ct  (Glut9) | Ct  (Gapdh) | Relative level | Mean | SE | p value |
| --- | --- | --- | --- | --- | --- | --- |
| Control rats | 28.73 | 17.40 | 0.58 | 1.00 | 0.21 |  |
|  | 27.20 | 16.81 | 1.12 |  |  |  |
|  | 26.27 | 16.09 | 1.30 |  |  |  |
| Hyperuricemic rats | 26.99 | 16.10 | 0.79 | 1.15 | 0.16 | 0.63 |
|  | 26.80 | 16.40 | 1.11 |  |  |  |
|  | 25.77 | 16.04 | 1.77 |  |  |  |
|  | 26.39 | 16.46 | 1.54 |  |  |  |
|  | 27.10 | 16.08 | 0.72 |  |  |  |
|  | 27.19 | 16.55 | 0.94 |  |  |  |

Unpaired Student’s t-test was used to analyze differences between groups.

**(J) Pept1**

|  | Ct  (Pept1) | Ct  (Gapdh) | Relative level | Mean | SE | p value |
| --- | --- | --- | --- | --- | --- | --- |
| Control rats | 29.89 | 19.60 | 1.20 | 1.00 | 0.20 |  |
|  | 30.20 | 18.92 | 0.61 |  |  |  |
|  | 29.67 | 19.37 | 1.19 |  |  |  |
| Hyperuricemic rats | 31.00 | 19.20 | 0.42 | 0.40 | 0.02 | 0.04 |
|  | 31.83 | 19.82 | 0.36 |  |  |  |
|  | 30.78 | 18.96 | 0.42 |  |  |  |

Unpaired Student’s t-test was used to analyze differences between groups.

**(K) Pept2**

|  | Ct  (Pept2) | Ct  (Gapdh) | Relative level | Mean | SE | p value |
| --- | --- | --- | --- | --- | --- | --- |
| Control rats | 22.99 | 19.60 | 0.99 | 1.00 | 0.20 |  |
|  | 21.85 | 18.92 | 1.36 |  |  |  |
|  | 23.34 | 19.37 | 0.66 |  |  |  |
| Hyperuricemic rats | 22.71 | 19.20 | 0.91 | 0.45 | 0.23 | 0.15 |
|  | 26.18 | 19.82 | 0.13 |  |  |  |
|  | 23.97 | 18.96 | 0.32 |  |  |  |

Unpaired Student’s t-test was used to analyze differences between groups.

**(L) Mdr1a**

|  | Ct  (Mdr1a) | Ct  (Gapdh) | Relative level | Mean | SE | p value |
| --- | --- | --- | --- | --- | --- | --- |
| Control rats | 27.18 | 19.60 | 0.99 | 1.00 | 0.05 |  |
|  | 26.61 | 18.92 | 0.92 |  |  |  |
|  | 26.81 | 19.37 | 1.09 |  |  |  |
| Hyperuricemic rats | 26.55 | 19.20 | 1.16 | 0.96 | 0.12 | 0.79 |
|  | 27.82 | 19.82 | 0.74 |  |  |  |
|  | 26.55 | 18.96 | 0.98 |  |  |  |

Unpaired Student’s t-test was used to analyze differences between groups.

**(M) Mdr1b**

|  | Ct  (Mdr1b) | Ct  (Gapdh) | Relative level | Mean | SE | p value |
| --- | --- | --- | --- | --- | --- | --- |
| Control rats | 27.94 | 19.60 | 0.62 | 1.00 | 0.23 |  |
|  | 26.05 | 18.92 | 1.42 |  |  |  |
|  | 27.07 | 19.37 | 0.96 |  |  |  |
| Hyperuricemic rats | 27.09 | 19.20 | 0.84 | 0.89 | 0.28 | 0.78 |
|  | 28.67 | 19.82 | 0.43 |  |  |  |
|  | 26.11 | 18.96 | 1.40 |  |  |  |

Unpaired Student’s t-test was used to analyze differences between groups.

**(N) Bcrp**

|  | Ct  (Bcrp) | Ct  (Gapdh) | Relative level | Mean | SE | p value |
| --- | --- | --- | --- | --- | --- | --- |
| Control rats | 24.10 | 19.60 | 0.93 | 1.00 | 0.05 |  |
|  | 23.34 | 18.92 | 0.98 |  |  |  |
|  | 23.64 | 19.37 | 1.09 |  |  |  |
| Hyperuricemic rats | 23.36 | 19.20 | 1.18 | 0.82 | 0.20 | 0.42 |
|  | 25.24 | 19.82 | 0.49 |  |  |  |
|  | 23.71 | 18.96 | 0.78 |  |  |  |

Unpaired Student’s t-test was used to analyze differences between groups.

Expression was estimated by the -ΔΔCt method and normalized to that of glyceraldehyde-3-phosphate dehydrogenase (Gapdh).
