# Supplementary material for: Changes of drug pharmacokinetics mediated by downregulation of kidney organic cation transporters Mate1 and Oct2 in a rat model of hyperuricemia
Source: PLoS One. 2019 Apr 5;14(4):e0214862. doi: 10.1371/journal.pone.0214862 (PMC6450621; doi:10.1371/journal.pone.0214862)
Supplement: S5 Table — (DOCX) [file pone.0214862.s005.docx]

**S5 Table. Plasma concentrations of metformin, cephalexin and creatinine and cumulative urinary excretions of metformin, cephalexin and creatinine in control and hyperuricemic rats (dataset of Fig 3).**

**(A) Plasma concentration of metformin**

| Time | Plasma concentration (µM) | | | | | | | | | | | p value |
| --- | --- | --- | --- | --- | --- | --- | --- | --- | --- | --- | --- | --- |
| (min) | Control rats | | | | |  | Hyperuricemic rats | | | | |  |
|  |  |  |  | Mean | SEM |  |  |  |  | Mean | SEM |  |
| 0 | 1272 | 750 | 1198 | 1073 | 163 |  | 676 | 796 | 923 | 798 | 71 | 0.20 |
| 0.5 | 871 | 571 | 817 | 753 | 92 |  | 578 | 780 | 862 | 740 | 84 | 0.92 |
| 2 | 385 | 293 | 304 | 328 | 29 |  | 267 | 336 | 407 | 337 | 40 | 0.87 |
| 5 | 159 | 154 | 164 | 159 | 3 |  | 86 | 154 | 193 | 144 | 32 | 0.67 |
| 15 | 67.3 | 58.6 | 67.1 | 64.4 | 2.9 |  | 61.1 | 53.1 | 68.6 | 60.9 | 4.5 | 0.55 |
| 30 | 37.0 | 33.2 | 34.5 | 34.9 | 1.1 |  | 30.0 | 54.0 | 44.1 | 42.7 | 7.0 | 0.33 |
| 60 | 14.4 | 13.0 | 12.8 | 13.4 | 0.5 |  | 20.6 | 26.4 | 27.5 | 24.9 | 2.1 | 0.007 |
| 120 | 3.94 | 5.06 | 3.26 | 4.09 | 0.52 |  | 3.71 | 4.25 | 5.60 | 4.52 | 0.56 | 0.60 |
| 240 | 1.11 | 1.90 | 0.87 | 1.29 | 0.31 |  | 1.92 | 1.49 | 1.90 | 1.77 | 0.14 | 0.24 |

Unpaired Student’s t-test was used to analyze differences between groups.

**(B) Plasma concentration of cephalexin**

| Time | Plasma concentration (µM) | | | | | | | | | | | p value |
| --- | --- | --- | --- | --- | --- | --- | --- | --- | --- | --- | --- | --- |
| (min) | Control rats | | | | |  | Hyperuricemic rats | | | | |  |
|  |  |  |  | Mean | SEM |  |  |  |  | Mean | SEM |  |
| 0 | 293 | 206 | 273 | 257 | 26 |  | 353 | 360 | 296 | 336 | 20 | 0.08 |
| 0.5 | 272 | 201 | 251 | 241 | 21 |  | 377 | 317 | 274 | 323 | 30 | 0.09 |
| 2 | 125 | 86 | 136 | 115 | 15 |  | 141 | 132 | 132 | 135 | 3 | 0.27 |
| 5 | 63.3 | 52.2 | 65.0 | 60.2 | 4.0 |  | 74.5 | 80.5 | 66.7 | 73.9 | 4.0 | 0.07 |
| 15 | 34.3 | 27.1 | 28.1 | 29.8 | 2.2 |  | 39.9 | 42.0 | 41.3 | 41.1 | 0.6 | 0.008 |
| 30 | 18.9 | 16.6 | 18.0 | 17.8 | 0.7 |  | 27.5 | 23.9 | 24.6 | 25.3 | 1.1 | 0.004 |
| 60 | 12.1 | 7.2 | 10.8 | 10.0 | 1.5 |  | 15.2 | 15.9 | 16.7 | 15.9 | 0.5 | 0.02 |
| 120 | 3.86 | 3.26 | 3.73 | 3.62 | 0.18 |  | 10.3 | 11.6 | 12.0 | 11.3 | 0.5 | 0.0001 |
| 240 | 1.18 | 0.58 | 2.31 | 1.36 | 0.51 |  | 8.22 | 8.31 | 9.61 | 8.72 | 0.45 | 0.0004 |

Unpaired Student’s t-test was used to analyze differences between groups.

**(C) Plasma concentration of creatinine**

| Time | Plasma concentration (µM) | | | | | | | | | | | p value |
| --- | --- | --- | --- | --- | --- | --- | --- | --- | --- | --- | --- | --- |
| (min) | Control rats | | | | |  | Hyperuricemic rats | | | | |  |
|  |  |  |  | Mean | SEM |  |  |  |  | Mean | SEM |  |
| 0.5 | 17.7 | 19.0 | 21.0 | 19.2 | 1.0 |  | 24.6 | 36.4 | 30.6 | 30.5 | 3.4 | 0.03 |
| 30 | 19.5 | 20.1 | 20.4 | 20.0 | 0.3 |  | 25.9 | 34.4 | 30.6 | 30.3 | 2.4 | 0.01 |
| 60 | 22.9 | 18.9 | 19.0 | 20.3 | 1.3 |  | 26.5 | 34.5 | 36.2 | 32.4 | 3.0 | 0.02 |
| 120 | 20.0 | 24.9 | 20.7 | 21.9 | 1.6 |  | 34.6 | 38.1 | 41.5 | 38.1 | 2.0 | 0.003 |
| 240 | 26.1 | 23.6 | 30.7 | 26.8 | 2.1 |  | 52.7 | 61.3 | 63.2 | 59.1 | 3.2 | 0.001 |

Unpaired Student’s t-test was used to analyze differences between groups.

**(D) Cumulative urinary excretion of metformin**

| Time | Urinary recovery (% of dose) | | | | | | | | | | | p value |
| --- | --- | --- | --- | --- | --- | --- | --- | --- | --- | --- | --- | --- |
| min | Control rats | | | | |  | Hyperuricemic rats | | | | |  |
|  |  |  |  | Mean | SEM |  |  |  |  | Mean | SEM |  |
| 10 | 15.5 | 4.4 | 33.4 | 17.8 | 8.4 |  | 15.5 | 16.7 | 11.6 | 14.6 | 1.5 | 0.73 |
| 20 | 26.0 | 21.2 | 46.7 | 31.3 | 7.8 |  | 26.9 | 26.1 | 20.0 | 24.3 | 2.2 | 0.44 |
| 30 | 43.9 | 27.9 | 51.8 | 41.2 | 7.0 |  | 35.7 | 31.6 | 27.5 | 31.6 | 2.4 | 0.27 |
| 60 | 57.8 | 44.6 | 64.4 | 55.6 | 5.8 |  | 46.0 | 41.1 | 37.0 | 41.4 | 2.6 | 0.09 |
| 120 | 61.5 | 63.3 | 72.5 | 65.7 | 3.4 |  | 56.1 | 49.6 | 47.8 | 51.1 | 2.5 | 0.03 |
| 240 | 75.1 | 72.0 | 76.4 | 74.5 | 1.3 |  | 67.5 | 63.2 | 59.1 | 63.3 | 2.4 | 0.02 |

Unpaired Student’s t-test was used to analyze differences between groups.

**(E) Cumulative urinary excretion of cephalexin**

| Time | Urinary recovery (% of dose) | | | | | | | | | | | p value |
| --- | --- | --- | --- | --- | --- | --- | --- | --- | --- | --- | --- | --- |
| min | Control rats | | | | |  | Hyperuricemic rats | | | | |  |
|  |  |  |  | Mean | SEM |  |  |  |  | Mean | SEM |  |
| 10 | 0.7 | 0.4 | 14.1 | 5.1 | 4.5 |  | 4.8 | 10.7 | 8.7 | 8.1 | 1.7 | 0.56 |
| 20 | 7.3 | 5.4 | 27.8 | 13.5 | 7.2 |  | 14.1 | 17.5 | 11.9 | 14.5 | 1.6 | 0.90 |
| 30 | 11.2 | 12.1 | 32.4 | 18.6 | 6.9 |  | 21.5 | 20.8 | 17.0 | 19.8 | 1.4 | 0.87 |
| 60 | 23.0 | 33.0 | 39.7 | 31.9 | 4.8 |  | 31.4 | 28.5 | 27.5 | 29.1 | 1.2 | 0.61 |
| 120 | 56.2 | 64.2 | 52.5 | 57.6 | 3.5 |  | 42.3 | 37.1 | 37.0 | 38.8 | 1.8 | 0.008 |
| 240 | 71.7 | 77.7 | 63.5 | 71.0 | 4.1 |  | 52.0 | 44.8 | 41.6 | 46.1 | 3.1 | 0.008 |

Unpaired Student’s t-test was used to analyze differences between groups.

**(F) Cumulative urinary excretion of creatinine**

| Time | Urinary excretion (mg) | | | | | | | | | | | p value |
| --- | --- | --- | --- | --- | --- | --- | --- | --- | --- | --- | --- | --- |
| min | Control rats | | | | |  | Hyperuricemic rats | | | | |  |
|  |  |  |  | Mean | SEM |  |  |  |  | Mean | SEM |  |
| 10 | 0.04 | 0.06 | 0.09 | 0.07 | 0.01 |  | 0.11 | 0.12 | 0.12 | 0.12 | 0.00 | 0.03 |
| 20 | 0.10 | 0.12 | 0.16 | 0.13 | 0.02 |  | 0.19 | 0.20 | 0.19 | 0.19 | 0.00 | 0.03 |
| 30 | 0.13 | 0.19 | 0.22 | 0.18 | 0.03 |  | 0.26 | 0.28 | 0.25 | 0.26 | 0.01 | 0.04 |
| 60 | 0.18 | 0.30 | 0.32 | 0.27 | 0.04 |  | 0.39 | 0.44 | 0.43 | 0.42 | 0.02 | 0.03 |
| 120 | 0.46 | 0.61 | 0.52 | 0.53 | 0.04 |  | 0.58 | 0.73 | 0.76 | 0.69 | 0.05 | 0.08 |
| 240 | 0.98 | 1.44 | 1.32 | 1.25 | 0.14 |  | 0.95 | 1.24 | 1.48 | 1.22 | 0.15 | 0.90 |

Unpaired Student’s t-test was used to analyze differences between groups.
