# Supplementary material for: Changes of drug pharmacokinetics mediated by downregulation of kidney organic cation transporters Mate1 and Oct2 in a rat model of hyperuricemia
Source: PLoS One. 2019 Apr 5;14(4):e0214862. doi: 10.1371/journal.pone.0214862 (PMC6450621; doi:10.1371/journal.pone.0214862)
Supplement: S6 Table — (DOCX) [file pone.0214862.s006.docx]

**S6 Table. Pharmacokinetic parameters of metformin, cephalexin and creatinine in control and hyperuricemic rats after intravenous administration of metformin or cephalexin (dataset of Table 2).**

**(A) Metformin**

|  |  | AUC_0-4_ | AUC_inf_ | CL_R_ | (CL_R_/f_u_)/CL_Inulin_ | Urinary Recovery | K_p, kidney_ |
| --- | --- | --- | --- | --- | --- | --- | --- |
|  |  | µM·min | µM·min | mL/min/kg |  | % of Dose/4 h |  |
| Control rats |  | 5891 | 5972 | 21.7 | 3.80 | 75.1 | 2.8 |
|  |  | 5208 | 5393 | 23.6 | 4.12 | 72.0 | 35.8 |
|  |  | 5490 | 5550 | 23.7 | 4.15 | 76.4 | 11.4 |
|  | Mean | 5530 | 5638 | 23.0 | 4.03 | 74.5 | 16.6 |
|  | SEM | 198 | 173 | 0.6 | 0.11 | 1.3 | 9.9 |
| Hyperuricemic rats |  | 4854 | 4997 | 23.7 | 5.20 | 67.5 | 54.1 |
|  |  | 6413 | 6513 | 16.8 | 3.69 | 63.2 | 94.7 |
|  |  | 7033 | 7159 | 14.3 | 3.14 | 59.1 | 122.2 |
|  | Mean | 6100 | 6223 | 18.3 | 4.01 | 63.3 | 90.3 |
|  | SEM | 648 | 641 | 2.8 | 0.62 | 2.4 | 19.8 |
| p value |  | 0.45 | 0.43 | 0.18 | 0.98 | 0.02 | 0.03 |

Unpaired Student’s t-test was used to analyze differences between groups.

**(B) Cephalexin**

|  |  | AUC_0-4_ | AUC_inf_ | CL_R_ | (CL_R_/f_u_)/CL_Inulin_ | Urinary Recovery | K_p, kidney_ |
| --- | --- | --- | --- | --- | --- | --- | --- |
|  |  | µM·min | µM·min | mL/min/kg |  | % of Dose/4 h |  |
| Control rats |  | 2874 | 2963 | 7.18 | 1.30 | 71.7 | 7.33 |
|  |  | 2188 | 2230 | 10.22 | 1.86 | 77.7 | 10.10 |
|  |  | 2825 | 3118 | 6.47 | 1.17 | 63.5 | 11.86 |
|  | Mean | 2629 | 2770 | 7.96 | 1.44 | 71.0 | 9.76 |
|  | SEM | 221 | 274 | 1.15 | 0.21 | 4.1 | 1.32 |
| Hyperuricemic rats |  | 4594 | 7174 | 3.26 | 0.74 | 52.0 | 14.8 |
|  |  | 4682 | 7076 | 2.75 | 0.63 | 44.8 | 14.6 |
|  |  | 4600 | 7893 | 2.60 | 0.59 | 41.6 | 15.8 |
|  | Mean | 4625 | 7381 | 2.87 | 0.65 | 46.1 | 15.1 |
|  | SEM | 28 | 258 | 0.20 | 0.05 | 3.1 | 0.4 |
| p value |  | 0.0009 | 0.0003 | 0.01 | 0.02 | 0.008 | 0.02 |

Unpaired Student’s t-test was used to analyze differences between groups.

**(C) Creatinine**

|  |  | AUC_0-4_ | CL_R_ | (CL_R_/f_u_)/CL_Inulin_ | K_p, kidney_ |
| --- | --- | --- | --- | --- | --- |
|  |  | µM·min | mL/min/kg |  |  |
| Control rats |  | 5255 | 8.8 | 1.31 | 8.8 |
|  |  | 5400 | 12.8 | 1.91 | 10.4 |
|  |  | 5463 | 11.1 | 1.65 | 11.3 |
|  | Mean | 5373 | 10.9 | 1.62 | 10.2 |
|  | SEM | 62 | 1.2 | 0.17 | 0.7 |
| Hyperuricemic rats |  | 8634 | 5.45 | 1.02 | 17.3 |
|  |  | 10284 | 5.88 | 1.10 | 12.0 |
|  |  | 10502 | 6.18 | 1.15 | 11.7 |
|  | Mean | 9807 | 5.84 | 1.09 | 13.7 |
|  | SEM | 590 | 0.21 | 0.04 | 1.8 |
| p value |  | 0.002 | 0.01 | 0.04 | 0.15 |

Unpaired Student’s t-test was used to analyze differences between groups.
