# Supplementary material for: Changes of drug pharmacokinetics mediated by downregulation of kidney organic cation transporters Mate1 and Oct2 in a rat model of hyperuricemia
Source: PLoS One. 2019 Apr 5;14(4):e0214862. doi: 10.1371/journal.pone.0214862 (PMC6450621; doi:10.1371/journal.pone.0214862)
Supplement: S7 Table — (DOCX) [file pone.0214862.s007.docx]

**S7 Table. Plasma concentration and cumulative urinary excretion of cephalexin after administration of high (10 mg/kg) and low (1 mg/kg) doses (dataset of Fig 4).**

**(A) Plasma concentration of cephalexin**

| Time | Plasma concentration (µM) | | | | | | | | | | | p value |
| --- | --- | --- | --- | --- | --- | --- | --- | --- | --- | --- | --- | --- |
| (min) | 1 mg/kg | | | | |  | 10 mg/kg | | | | |  |
|  |  |  |  | Mean | SEM |  |  |  |  | Mean | SEM |  |
| 0 | 30.2 | 30.0 | 20.0 | 26.7 | 3.4 |  | 293 | 206 | 273 | 257 | 26 | 0.001 |
| 0.5 | 21.8 | 23.3 | 17.5 | 20.9 | 1.7 |  | 272 | 201 | 251 | 241 | 21 | 0.0005 |
| 2 | 9.2 | 12.0 | 11.2 | 10.8 | 0.8 |  | 125 | 86 | 136 | 115 | 15 | 0.002 |
| 5 | 7.23 | 9.06 | 7.53 | 7.94 | 0.57 |  | 63.3 | 52.2 | 65.0 | 60.2 | 4.0 | 0.0002 |
| 15 | 4.93 | 5.96 | 5.12 | 5.34 | 0.32 |  | 34.3 | 27.1 | 28.1 | 29.8 | 2.2 | 0.0004 |
| 30 | 3.18 | 8.89 | 4.03 | 5.37 | 1.78 |  | 18.9 | 16.6 | 18.0 | 17.8 | 0.7 | 0.003 |
| 60 | 3.19 | 3.33 | 2.74 | 3.09 | 0.18 |  | 12.1 | 7.2 | 10.8 | 10.0 | 1.5 | 0.01 |
| 120 | 2.43 | 1.54 | 1.09 | 1.69 | 0.39 |  | 3.86 | 3.26 | 3.73 | 3.62 | 0.18 | 0.01 |
| 240 | 1.15 | 1.64 | 0.45 | 1.08 | 0.35 |  | 1.18 | 0.58 | 2.31 | 1.36 | 0.51 | 0.67 |

Unpaired Student’s t-test was used to analyze differences between groups.

**(B) Cumulative urinary excretion of cephalexin**

| Time | Urinary recovery (% of dose) | | | | | | | | | | | p value |
| --- | --- | --- | --- | --- | --- | --- | --- | --- | --- | --- | --- | --- |
| min | 1 mg/kg | | | | |  | 10 mg/kg | | | | |  |
|  |  |  |  | Mean | SEM |  |  |  |  | Mean | SEM |  |
| 10 | 0.4 | 2.9 | 3.8 | 2.3 | 1.0 |  | 0.7 | 0.4 | 14.1 | 5.1 | 4.5 | 0.59 |
| 20 | 6.8 | 4.2 | 10.5 | 7.2 | 1.8 |  | 7.3 | 5.4 | 27.8 | 13.5 | 7.2 | 0.44 |
| 30 | 28.3 | 12.4 | 18.5 | 19.7 | 4.6 |  | 11.2 | 12.1 | 32.4 | 18.6 | 6.9 | 0.89 |
| 60 | 32.4 | 22.4 | 27.1 | 27.3 | 2.9 |  | 23.0 | 33.0 | 39.7 | 31.9 | 4.8 | 0.46 |
| 120 | 34.9 | 33.2 | 34.0 | 34.0 | 0.5 |  | 56.2 | 64.2 | 52.5 | 57.6 | 3.5 | 0.003 |
| 240 | 40.8 | 37.4 | 36.9 | 38.3 | 1.2 |  | 71.7 | 77.7 | 63.5 | 71.0 | 4.1 | 0.002 |

Unpaired Student’s t-test was used to analyze differences between groups.
