# Supplementary material for: Changes of drug pharmacokinetics mediated by downregulation of kidney organic cation transporters Mate1 and Oct2 in a rat model of hyperuricemia
Source: PLoS One. 2019 Apr 5;14(4):e0214862. doi: 10.1371/journal.pone.0214862 (PMC6450621; doi:10.1371/journal.pone.0214862)
Supplement: S8 Table — (DOCX) [file pone.0214862.s008.docx]

**S8 Table. Pharmacokinetic parameters of cephalexin in control rats after intravenous administration of cephalexin (dataset of Table 3).**

| Dose |  | AUC_0-4_ | AUC_inf_ | CL_tot_ | CL_R_ | CL_cr_ | (CL_R_/f_u_)/CL_Inulin_ | Urinary Recovery |
| --- | --- | --- | --- | --- | --- | --- | --- | --- |
|  |  | µM·min | µM·min | mL/min/kg | mL/min/kg | mL/min/kg |  | % of Dose/4 h |
| 1 mg/kg |  | 665 | 864 | 3.33 | 1.74 | 8.69 | 0.32 | 40.1 |
|  |  | 788 | 1280 | 2.25 | 1.43 | 7.36 | 0.26 | 39.2 |
|  |  | 514 | 560 | 5.14 | 2.46 | 6.93 | 0.45 | 44.0 |
|  | Mean | 656 | 901 | 3.57 | 1.88 | 7.66 | 0.34 | 41.1 |
|  | SEM | 79 | 209 | 0.84 | 0.31 | 0.53 | 0.06 | 1.5 |
| 10 mg/kg |  | 2874 | 2963 | 9.7 | 7.18 | 8.8 | 1.30 | 71.7 |
|  |  | 2188 | 2230 | 12.9 | 10.22 | 12.8 | 1.86 | 77.7 |
|  |  | 2825 | 3118 | 9.2 | 6.47 | 11.1 | 1.17 | 63.5 |
|  | Mean | 2629 | 2770 | 10.6 | 7.96 | 10.9 | 1.44 | 71.0 |
|  | SEM | 221 | 274 | 1.2 | 1.15 | 1.2 | 0.21 | 4.1 |
| p value |  | 0.001 | 0.006 | 0.008 | 0.007 | 0.06 | 0.007 | 0.002 |

Unpaired Student’s t-test was used to analyze differences between groups.
