# Supplementary material for: Changes of drug pharmacokinetics mediated by downregulation of kidney organic cation transporters Mate1 and Oct2 in a rat model of hyperuricemia
Source: PLoS One. 2019 Apr 5;14(4):e0214862. doi: 10.1371/journal.pone.0214862 (PMC6450621; doi:10.1371/journal.pone.0214862)
Supplement: S9 Table — (DOCX) [file pone.0214862.s009.docx]

**S9 Table. Protein expression level of kidney Mate1 in control and hyperuricemic rats (dataset of Fig 5).**

|  | Mate1 | Gapdh | % of control | | SEM | p value |
| --- | --- | --- | --- | --- | --- | --- |
|  | Density | Density |  | Mean |  |  |
| Control rats | 3751 | 4106 | 101 | 100.0 | 6.7 |  |
|  | 2707 | 3389 | 88 |  |  |  |
|  | 3576 | 3539 | 111 |  |  |  |
| Hyperuricemic rats | 2577 | 4431 | 64 | 60.7 | 1.7 | 0.005 |
|  | 2014 | 3752 | 59 |  |  |  |
|  | 3563 | 6685 | 59 |  |  |  |

Unpaired Student’s t-test was used to analyze differences between groups.
