# Supplementary material for: Changes of drug pharmacokinetics mediated by downregulation of kidney organic cation transporters Mate1 and Oct2 in a rat model of hyperuricemia
Source: PLoS One. 2019 Apr 5;14(4):e0214862. doi: 10.1371/journal.pone.0214862 (PMC6450621; doi:10.1371/journal.pone.0214862)
Supplement: S10 Table — (DOCX) [file pone.0214862.s010.docx]

**S10 Table. Effects of oxonic acid, adenine and uric acid on uptake of MPP^+^ by MATE1-expressing cells (dataset of Fig 6).**

|  |  | Control | Cimetidine |  | Oxonic acid | |  | Adenine | |  | Uric acid | |
| --- | --- | --- | --- | --- | --- | --- | --- | --- | --- | --- | --- | --- |
|  |  |  | 100 µM |  | 50 µM | 500 µM |  | 50 µM | 500 µM |  | 50 µM | 500 µM |
| C/M ratio |  | 18.8 | 9.40 |  | 18.7 | 19.2 |  | 19.1 | 14.1 |  | 20.9 | 25.2 |
| (µL/mg protein) |  | 19.3 | 10.25 |  | 13.1 | 28.0 |  | 14.6 | 13.0 |  | 21.9 | 17.6 |
| Mock cells |  | 23.5 | 9.69 |  | 12.3 | 20.9 |  | 15.4 | 20.0 |  | 19.7 | 29.8 |
|  | Mean | 20.5 | 9.78 |  | 14.7 | 22.7 |  | 16.4 | 15.7 |  | 20.8 | 24.2 |
|  | SEM | 1.5 | 0.25 |  | 2.0 | 2.7 |  | 1.4 | 2.2 |  | 0.6 | 3.6 |
| C/M ratio |  | 230 | 18.8 |  | 183 | 235 |  | 139 | 177 |  | 195 | 182 |
| (µL/mg protein) |  | 202 | 36.0 |  | 177 | 173 |  | 209 | 143 |  | 166 | 236 |
| MATE1-expressing |  | 169 | 18.8 |  | 232 | 190 |  | 174 | 196 |  | 252 | 198 |
| cells | Mean | 200 | 24.5 |  | 197 | 199 |  | 174 | 172 |  | 204 | 205 |
|  | SEM | 18 | 5.7 |  | 18 | 19 |  | 20 | 15 |  | 25 | 16 |
| % of Control |  | 116.6 | 5.0 |  | 93.3 | 118.1 |  | 68.3 | 89.5 |  | 96.8 | 87.8 |
|  |  | 100.7 | 14.6 |  | 90.1 | 83.7 |  | 107.0 | 71.0 |  | 81.0 | 117.9 |
|  |  | 82.7 | 5.0 |  | 121.0 | 92.8 |  | 87.8 | 100.5 |  | 128.4 | 96.4 |
|  | Mean | 100.0 | 8.2 |  | 101.5 | 98.2 |  | 87.7 | 87.0 |  | 102.0 | 100.7 |
|  | SEM | 9.8 | 3.2 |  | 9.8 | 10.3 |  | 11.1 | 8.6 |  | 13.9 | 9.0 |
| p value |  |  | 0.00004 |  | 1.00 | 1.00 |  | 0.91 | 0.89 |  | 1.00 | 1.00 |

Dunnett's test was used for uptake study to compare the control group to groups treated with drugs.

Percentages of control were calculated as the ratio of MATE1-mediated uptake of MPP^+^ that obtained after subtraction of the uptake of MPP^+^ by mock cells in compound treated groups to that in control group.
